# Synthesis of Novel 3,6-Dithienyl Diketopyrrolopyrrole Dyes by Direct C-H Arylation

**DOI:** 10.3390/molecules25102349

**Published:** 2020-05-18

**Authors:** Amsalu Efrem Yemene, Vishwesh Venkatraman, David Moe Almenningen, Bård Helge Hoff, Odd Reidar Gautun

**Affiliations:** Department of Chemistry, Norwegian University of Science and Technology (NTNU), N-7491 Trondheim, Norway; amsalu.e.yemene@ntnu.no (A.E.Y.); Vishwesh.Venkatraman@ntnu.no (V.V.); David.Moe.Almenningen@ntnu.no (D.M.A.)

**Keywords:** direct C-H arylation, dye-sensitized solar cells, diketopyrrolopyrrole, phenothiazine, triarylamine, dye

## Abstract

Direct C-H arylation coupling is potentially a more economical and sustainable process than conventional cross-coupling. However, this method has found limited application in the synthesis of organic dyes for dye-sensitized solar cells. Although direct C-H arylation is not an universal solution to any cross-coupling reactions, it efficiently complements conventional sp^2^−sp^2^ bond formation and can provide shorter and more efficient routes to diketopyrrolopyrrole dyes. Here, we have applied palladium catalyzed direct C-H arylation in the synthesis of five new 3,6-dithienyl diketopyrrolopyrrole dyes. All prepared sensitizers display broad absorption from 350 nm up to 800 nm with high molar extinction coefficients. The dye-sensitized solar cells based on these dyes exhibit a power conversion efficiency in the range of 2.9 to 3.4%.

## 1. Introduction

**Figure 1:**
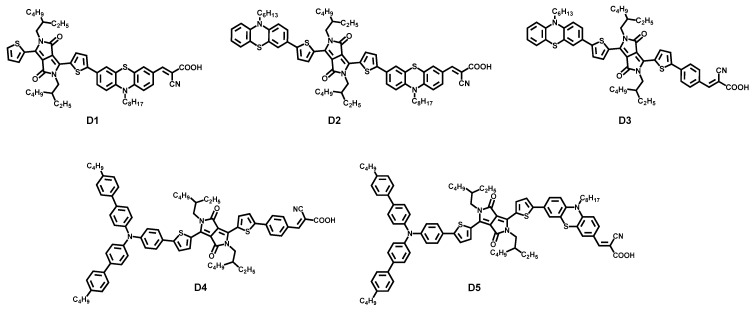
Chemical structures of the target dyes **D1**–**D5**.

The development of metal-free organic dyes with tunable optoelectronic properties is of importance among others, in the field of dye-sensitized solar cells (DSSCs). These dyes have interesting features like low cost, flexible molecular design, high molar absorption coefficients, and efficient intramolecular charge transfer (ICT) [1,2,3]. metal-free organic dyes are conventionally synthesized via sequential transformation of building blocks using transition metal-catalyzed cross-coupling reactions, such as Suzuki-Miyaura [4,5] and Migita-Kosugi-Stille couplings [6,7,8]. Despite their great versatility, these protocols often require the preparation of more advanced and expensive or toxic organometallic functionalized units such as arylene boronic acid/boronic esters for Suzuki and aryl stannanes for Stille-type reactions. Recently, a new C-C bond forming methodology called direct C-H arylation has entered the toolbox of chemists, which involves the coupling of an aryl halide directly with another arene without the need for pre-functionalization of C-H bonds. Direct C-H arylation has been extensively employed in the synthesis of conjugated polymers for organic photovoltaics (OPVs) and organic field effect transistor applications (OFETs) [9,10,11], and has been used for preparation of metal-free organic dyes [12,13,14,15,16,17]. For the future commercialization of technologies with organic dyes, implementing economically and environmentally sound synthetic protocol is of immense importance. Direct C-H arylation can in this respect be highly useful as it can enable the synthesis of organic dyes in fewer steps. Moreover, direct C-H arylation complements established cross-coupling methodology by providing alternative synthetic routes. In order to display the potential of direct C-H arylation in the synthesis of dyes, we herein report the preparation of five new 3,6-dithienyl diketopyrrolopyrrole (3,6-dithienyl-DPP) dyes (see Figure 1).

## 2. Results and Discussion

Diketopyrrolopyrrole (DPP) was selected as the core building block for our dyes as it has a planar framework, which allows efficient charge transport, and broad and intense spectral absorption in the visible and NIR region. DPP-based sensitizers have been reported for use in DSSCs, but all were synthesized using conventional coupling methods [18,19,20,21,22,23,24,25,26,27,28,29]. Thus, it was of interest to investigate direct C-H arylation in the synthesis of this dye class.

The challenge with DPP dyes is their low solubility and tendency to aggregate [26,29]. Therefore, the dyes were decorated with solubilizing alkyl chains. Moreover, the incorporation of phenothiazine unit which has a “butterfly” conformation in the ground state [30,31], should be convenient for improving solubility and decreased intermolecular π-π interaction. Phenothiazine and phenyl were used instead of thiophene and furan as the π-bridge between the main chromophore and the anchoring group. The downside of this design is a lager dihedral angle between the chromophore and the linker, that may be outweighed by a higher solubility.

### 2.1. Dye Synthesis by Direct C-H Arylations

**Scheme 1:**
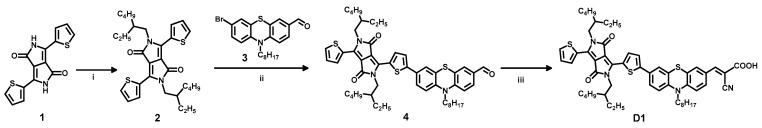
Synthesis route of dye **D1**. *Reagents and Conditions*: (i) 2-Ethylhexyl bromide, K_2_CO_3_, DMF, 145 °C, 15 h, 33% yield; (ii) Pd_2_(dba)_3_, (*o*-MeOPh)_3_P, PivOH, Cs_2_CO_3_, toluene, 120 °C, overnight, 63% yield; (iii) 2-cyanoacetic acid, acetonitrile, piperidine 80 °C, 2 h, 89% yield.

Dyes **D1** and **D2** both have phenothiazine as a *|D*-linker. The synthetic route to dye **D1** is depicted in Scheme 1. Firstly, *N*-alkylation of commercially available 3,6-dithienyl-DPP (**1**) was performed using 2-ethylhexylbromide as alkylating agent, which provided *N,N*-dialkylated **2** in 33% yield. Both *N,O*- and *O*-alkylated DPP were observed as by-product by us and others [32,33], which appers to be overseen in some previous reports. Due to the chiral racemicnature of the alkyl side chains, compound **2** and all later derivatives are obtained as an inseparable mixture of diastereomers.

Derivative **2** was then subjected to a C-H arylation with the phenothiazine **3** in dry toluene at 120 °C using Pd_2_(dba)_3_ as catalyst and (o-MeOPh)_3_P as ligand. This afforded the dye precursor **4** in 64% isolated yield. Although, the yield may not seem impressive, a rather difficult functionalization of the thienyl moiety is avoided. A Knoevenagel condensation on **4** with 2-cyanoacetic acid promoted by piperidine resulted in dye **D1** (89% yield).

**Scheme 2:**
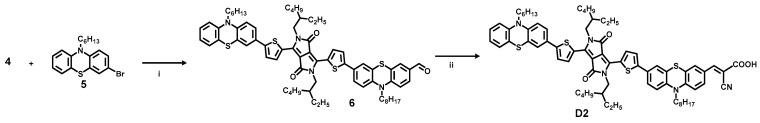
Synthesis route to dye **D2**. *Reagents and Conditions*: (i) Pd_2_(dba)_3_, (*o*-MeOPh)_3_P, PivOH, Cs_2_CO_3_, toluene, 120 °C, 2 h, 49% yield; (ii) 2-cyanoacetic acid, acetonitrile, piperidine 80 °C, 2 h, 85% yield.

To arrive at dye **D2** a sequential direct C-H arylation was performed in which **4** was further reacted with 3-bromo-10-hexyl-10*H*-phenothiazine (**5**) to afford **6** in 43% yield, see Scheme 2. Finally, dye **D2** was obtained in 85% isolated yield by a Knoevenagel condensation.

We also attempted to prepare the pinacol borane ester of **3** by palladium catalyzed borylation, but this transformation was unsuccessful, and only the corresponding phenothiazine alcohol was observed. Although protection of the aldehyde function will solve this problem, it appears that routes based on Suzuki cross-coupling has drawbacks. In contrast direct C-H arylation has the clear advantage of fewer steps without the need to pre-functionalize phenothiazines (borylation or aldehyde protection).

**Scheme 3:**
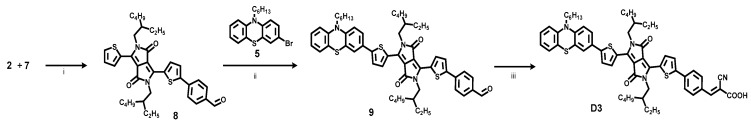
Synthesis of dye **D3**. *Reagents and Conditions*: (i) Pd_2_(dba)_3_, (o-MeOPh)_3_P, PivOH, Cs_2_CO_3_, toluene, 120 °C, overnight, 64% yield; (ii) Pd_2_(dba)_3_, (o-MeOPh)_3_P, PivOH, Cs_2_CO_3_, toluene, 120 °C, overnight, 54% yield; (ii) 2-cyanoacetic acid, acetonitrile:CHCl_3_ (4:1 *v*/*v*), piperidine 80 °C, 3 h, 80% yield.

The synthesis of dye **D3** was planned with a phenothiazine as donor moiety (Scheme 3). Its synthesis was performed with two sequential direct C-H arylations, in a similar way as for dye **D2**. The 3,6-dithienyl-DPP **2** was first coupled with 4-bromobenzaldehyde (**7**) to give compound **8** in 64% yield, followed by the second direct C-H arylation with 3-bromo-10-hexyl-*10H*-phenothiazine (**5**) to give **9** in 54% yield. Finally, a Knoevenagel condensation resulted in dye **D3** in 80% yield. 

From the study of Holcombe et al. [26] it is clear that dye **D3** in principal also could be prepared by two Suzuki cross-couplings, as they effectively brominated derivative **8** in 89% yield. However, as compared to the C-H arylation strategy presented in Scheme 3, additionally two bromination and two borylation steps would be required. In this context C-H arylation provides a much shorter synthetic route.

**Scheme 4:**
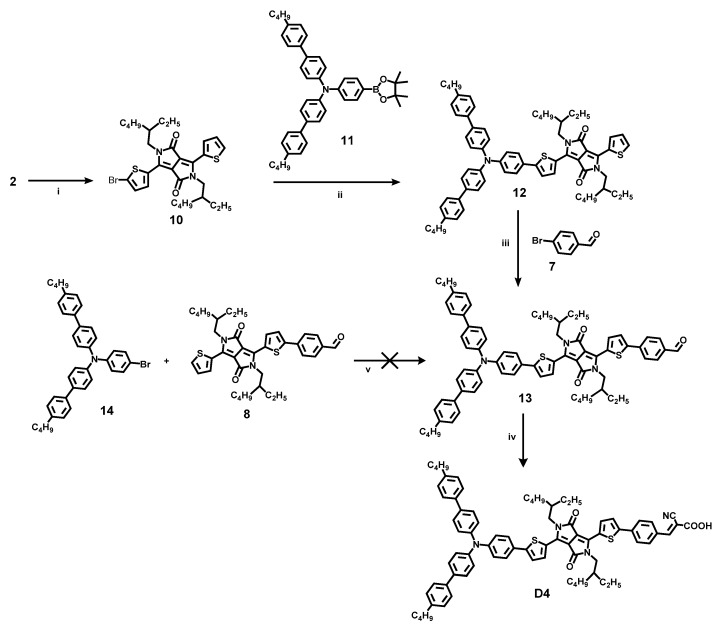
Synthesis of **D4**. *Reagents and Conditions*: (i) NBS, CHCl_3_, 22 °C overnight, 46% yield; (ii) Pd(PPh)_3_, K_2_CO_3_, H_2_O, EtOH, toluene, 110 °C, 24 h, 60% yield; (iii) Pd_2_(dba)_3_, (o-MeOPh)_3_P, Cs_2_CO_3_, toluene, 120 °C, overnight, 67% yield; (iv) 2-cyanoacetic acid, acetonitrile:CHCl_3_ (4:1 *v*/*v*), piperidine, 80 °C, 3 h, 97% yield; (v) Pd_2_(dba)_3_, (o-MeOPh)_3_P, PivOH, Cs_2_CO_3_, toluene, 120 °C, 24 h, failed reaction.

In contrary to **D2** and **D3**, the dyes **D4** and **D5** both contain a triarylamine donor group. The synthesis of dye **D4** is shown in Scheme 4. A shortcut synthetic route to **D4** was first evaluated employing direct C-H arylation between 3,6-dithienyl-DPP derivative **8** and the brominated triarylamine **14**. However, the reaction mixture was very complex, possibly due to homocoupling and poor C-H selectivity, thus the target aldehyde **13** could not be isolated. Obviously, the rates of the different mechanistical steps does not match as well as for couplings with brominated phenothiazines. Although, this step may be improved, for instance, by employing the corresponding triaryl iodide and Cu-catalysis [16], here, we have employed a Suzuki approach. The synthesis commences with a monobromination of **2** using NBS to afford **10** in 46% yield, followed by Suzuki cross-coupling with the triarylamine pinacol borane **11** giving the advanced intermediate **12** in 60% yield. Further, direct C-H arylation on **12** with 4-bromobenzaldehyde (**7**) afforded the dye precursor **13** in 67% yield, before a Knoevenagel condensation concluded the synthesis of **D4**. The synthesis of dye **D4** clearly shows the complementarity of direct C-H arylation and Suzuki cross-coupling. Although the Suzuki approach requires additional steps, it safely delivers the target material.

**Scheme 5:**
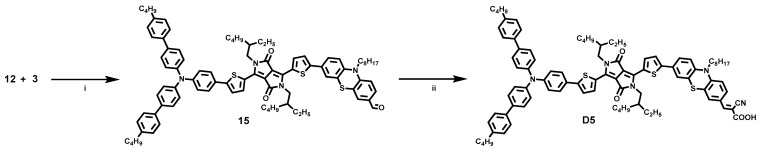
Synthesis of dye **D5**. *Reagents and Conditions*: (i) Pd_2_(dba)_3_, (o-MeOPh)_3_P, Cs_2_CO_3_, toluene, 120 °C, overnight, 77% yield; (ii) 2-cyanoacetic acid, acetonitrile: CHCl_3_ (4:1 *v*/*v*), piperidine, 80 °C, 3 h, 80% yield.

Dye **D5** has the same donor moiety as dye **D4**, and the synthesis could be started with the advanced DPP intermediate **12** already prepared (Scheme 4). It is well noted that the phenothiazine derivative **3** was a good coupling partner in direct C-H arylations in the previous reactions, and indeed the aldehyde **15** was obtained in 77% yield following reaction between **12** and **3** (Scheme 5). Dye **D5** was then easily isolated in good yield after reaction with cyanoacetic acid.

Overall, the synthetic work towards dyes **D1**–**D5** shows that direct C-H arylation provides a useful complement to other cross-coupling methodologies in synthesis of 3,6-dithienyl diketopyrrolopyrrole dyes and could shorten the synthesis route and increase the overall yield. However, challenges can also be encountered as was evident when employing brominated triarylamines in direct C-H arylations.

### 2.2. Photophysical and Electrochemical Properties

**Figure 2:**
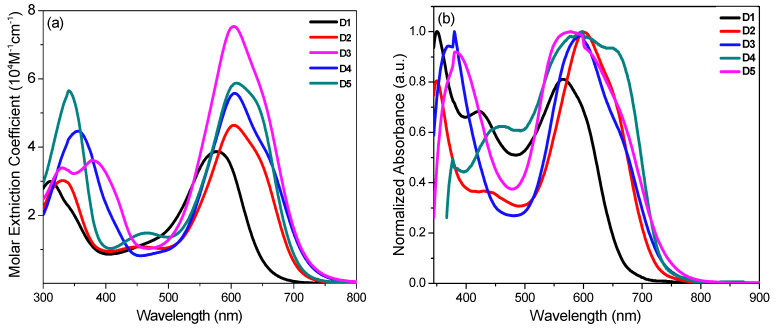
UV-Visible spectra in dichloromethane solution (2 × 10^−5^ M) (**a**), on TiO_2_ (**b**).

The absorption spectra of the five dyes, **D1**–**D5** in diluted dichloromethane (CH_2_Cl_2_) solution and the normalized absorption spectra for all dyes adsorbed on TiO_2_ films are depicted in Figure 2a,b, respectively. The photophysical and electrochemical properties are summarized in Table 1. All dyes show similar broad absorption spectra covering a wide range of the UV-visible region and almost up to the NIR region with a pronounced dip at around 450 nm. The dyes exhibit two major absorption bands, one appearing < 450 nm due to localized aromatic π-π* transitions and one > 500 nm originating from intramolecular charge transfer (ICT) between the donor and acceptor moiety of the dyes. The λ_max_ in CH_2_Cl_2_ solution for **D2** to **D5** is almost equal, which is in the range of 604–608 nm, whereas 576 nm is seen for **D1**. The red-shifted λ_max_ for **D2** to **D5** can be ascribed to an increased ICT due to the additional donor moiety. As shown in Table 1, all dyes have a high molar absorption extinction coefficient (ε) especially **D3** (7.548 × 10^4^ M^−1^ cm^−1^). After anchoring on TiO_2_ (Figure 2b), all dyes showed blue-shifted (hypsochromism) and broadened absorption spectra compared to the spectra measured in solution, which might be ascribed to the deprotonation of the carboxylic acid [34] and aggregation [35]. In particular, the ICT band for **D3** hypsochomically shifted by 26 nm, which is larger than the other dyes. The possible reason might be a stronger tendency to form aggregates.

The electrochemical properties of the five dyes were investigated by cyclic voltammetry (CV) in acetonitrile solution containing 0.1 M tetra-n-butylammonium hexafluorophosphate (TBAPF_6_) as the supporting electrolyte (see Figure S2, Supporting information). The redox potentials were estimated by the inflection potential, which has shown to be the best estimate of E^(0)^ in the cases of complete chemical irreversibility [36], and externally calibrated by the ferrocenium/ferrocene (Fc^+^/Fc) redox couple (0.42 V vs. NHE). The derived energy levels are tabulated in Table 1. The highest occupied molecular orbital (HOMO) level, which corresponds to the first oxidation potential for dyes **D1**–**D5** are determined to be 1.12, 1.02, 0.98, 1.04 and 1.02 V (vs NHE), respectively, which are more positive than the I^−^/I_3_^−^ redox couples (~0.42 V vs. NHE).

**Figure 3:**
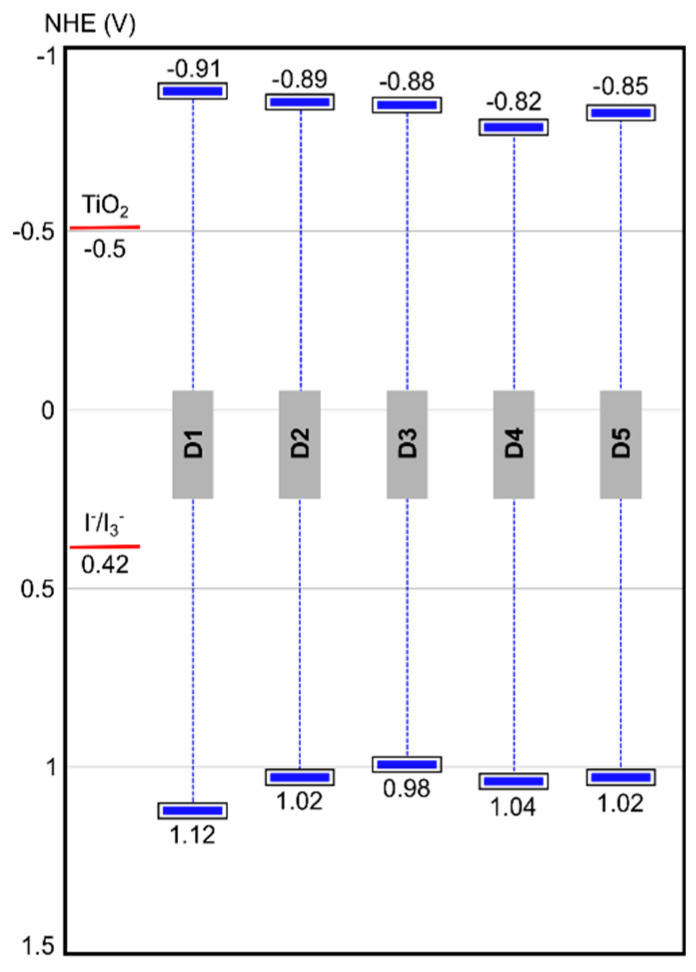
HOMO and LUMO levels of **D1**–**D5**.

This indicates that reduction of the oxidized dyes is thermodynamically feasible (see Figure 3). The lowest unoccupied molecular orbital (LUMO) levels were calculated by subtraction of E_0−0_, from HOMO. Thus, the LUMO levels for **D1**, **D2**, **D3**, **D4** and **D5** dyes were calculated to be −22,120.91, −0.89, −0.88, −0.82 and −0.85 V, respectively, indicating sufficient driving force for electron injection from the exited state of the dyes to the TiO_2_ films (see Figure 3). 

### 2.3. Photovoltaic Properties

**Figure 4:**
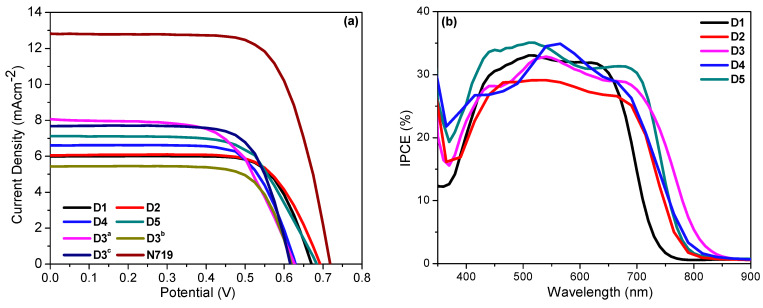
(**a**) Photocurrent density-voltage (J-V) curves and (**b**) Incident photon-to–electron conversion efficiency (IPCE) spectra of the dyes **D1** to **D5**.

The photovoltaic properties of **D1**–**D5** dyes were investigated by fabricating conventional DSSCs using the I^−^/I_3_^−^ redox couple as the electrolyte. The TiO_2_ photoanodes were initially stained in a binary mixture of THF:acetonitrile; 0.57:0.47 *v*/*v*) using a dye concentration of 0.5 mM and 5 mM chenodeoxycholic acid (CDCA). Figure 4a shows the photocurrent density-voltage (J-V) curve of the dyes measured under global AM 1.5G sunlight with irradiance of 100 mWcm^−2^ at 298 K. All the device parameters are summarized in Table 2. 

Overall, the performance of the five dyes in terms of power conversion efficiency (PCE) was quite similar ranging from 2.83–3.35%. The dyes with phenothiazine as π-linker (**D1**, **D2** and **D5**) had a higher open circuit voltage (V_oc_) than those with phenyl. The highest short circuit current (J_sc_) was seen for dye **D3** having a phenothiazine donor and phenyl as π-spacer. Dye **D3** was also evaluated using THF as staining solvent with and without CDCA. In the absence of CDCA the PCE dropped to 2.47%, while with CDCA an increased fill factor was seen giving a PCE of 3.41%.

The mediocre performance of the devices is also evident from the IPCE spectra (Figure 4b), which do not exceed 35%. It is apparent that the low efficiencies are due to a suboptimal photon to electron conversion process. DFT calculations (see Table S1, Supporting Information) also indicate that π-electron delocalization in the LUMO level, especially for dyes containing phenothiazine as a π–linker, is poor, which might make charge separation in the excited state more difficult. 

The performance of these dyes in DSSC might be improved by optimizing the staining conditions, the amount of CDCA, by co-sensitization, and by employing electrolytes providing higher voltage. However, it appears that the use of symmetrical 3,6-dithienyl–DPP as a design concept has clear limitations, as rather poor PCE has been reported in literature [26]. In contrast, higher efficiency has been obtained using 3,6-diphenyl–DPP [19,23] and asymmetrical 3,6-diaryl-DPP as the core building block of the dyes [18,19,28,29,37]. Therefore, further efforts towards DPP based dyes for DSSC should proceed using these or other non-symmetrical DPP building blocks. However, the dyes at hand might find alternative application for instance as optical filters.

## 3. Materials and Methods 

### 3.1. General 

Unless stated otherwise, all chemicals and solvents were obtained from commercial sources and used without further purification. 7-Bromo-10-octyl-10*H*-phenothiazine-3-carbaldehyde (**3**) was prepared in a previous study [38], while 3-bromo-10-hexyl-10*H*-phenothiazine (**5**) was prepared as described by Wang et al. [39] NMR spectroscopy (^1^H and ^13^C) was recorded on 400 and 600 MHz instruments (Bruker, Billerica, MA, USA) and all chemical shifts are reported relative to the respective solvent signals. Mass determination was performed on a Synapt G2–S QTOF instrument (Waters, Milford, MA, USA) in positive and negative modes. UV–Vis spectra were recorded on a U–1900 instrument (Hitachi, Chiyoda City, Japan) using quartz cuvettes for the solution samples, while fluorescence spectroscopy was recorded on a FS5 spectrofluorometer (Edinburg Instruments Ltd., Livingstone, UK). Infrared spectroscopy was recorded on an FTIR Nexus FT–IR spectrophotometer (Thermo Nicolet, Champaign, IL, USA).

### 3.2. Synthesis of Dye D1

#### 3.2.1. 5-Diethylhexyl-3,6-dithiophen-2-ylpyrrolo-[3,4-c]pyrrole-1,4-dione (**2**)

Compound **1** (1.00 g, 3.30 mmol, 1 eq.) and anhydrous potassium carbonate (1.84 g, 13.3 mmol, 4 eq.) were dissolved into dry *N*,*N*−dimethylformamide (25 mL) in a two–neck round flask and heated to 145 °C under nitrogen protection. 2-ethylhexyl bromide (2.96 g, 15.3 mmol, 4.6 eq.) was injected in one portion by syringe. The reaction mixture was stirred for 20 h at 145 °C, then the solution was cooled to room temperature (rt.) and poured into ice water (50 mL). The aqueous layer was extracted with CH_2_Cl_2_ (3 × 50 mL). The combined organic extracts were dried over Na_2_SO_4_, filtered and concentrated under reduced pressure. The resulting material was purified by silica gel column chromatography using hexane:EtOAc (9:1) leading to **2** (0.57 g, 1.08 mmol, 33%) as a black red solid. ^1^H-NMR (400 MHz, CDCl_3_, 298 K): *δ* 8.91 (d, *J* = 8.9 Hz, 2H), 7.62 (d, *J* = 7.6 Hz, 2H), 7.27 (d, *J* = 7.3 Hz, 2H), 4.03–4.00 (m, 4H), 1.88–1.85 (m, 2H), 1.38–1.24 (m, 16H), 0.89–0.83 (m, 12H). ^13^C-NMR (100 MHz, CDCl_3_, 298 K): *δ* 161.7, 140.3, 135.2, 130.5, 129.8, 128.4, 107.8, 77.3, 77.0, 76.7, 45.8, 39.0, 30.2, 30.1, 28.3, 23.5, 23.0, 14.0, 10.4. HRMS (TOF ASAP+, *m/z*): observed 525.2600, calculated for C_30_H_41_N_2_O_2_S_2_ [M + H]^+^ 525.2604. The ^1^H-NMR data was in accordance with reported literature [40]. 

#### 3.2.2. 7-(5-(2,5-Bis(2-ethylhexyl)-3,6-dioxo-4-(thiophen-2-yl)-2,3,5,6-tetrahydropyrrolo[3,4-c]pyrrol-1-yl)-thiophen-2-yl)-10-octyl-10*H*-phenothiazine-3-carbaldehyde (**4**)

In a Schlenk tube 7-bromo-10-octyl-10*H*-phenothiazine-3-carbaldehyde (**3**, 29.8 mg, 0.071 mmol, 1.0 eq.), **2** (60 mg, 0.114 mmol, 1.6 eq.), Pd_2_(dba)_3_ (8.24 mg, 0.009 mmol, 12 mol%), (*o*-MeOPh)_3_P (6.34 mg, 0.048 mmol, 24 mol%), Cs_2_CO_3_ (27.3 mg, 0.084 mmol, 1.1 eq.), pivalic acid (1.6 mg, 0.015 mmol, 0.2 eq.) were added under nitrogen and then dissolved in dry toluene (1.0 mL). The reaction mixture was stirred for 18 h at 120 °C. After cooling to rt. the reaction mixture was poured in water (20 mL). The aqueous layer was extracted with CH_2_Cl_2_ (3 × 15 mL). The combined organic extracts were dried over Na_2_SO_4_, filtered and concentrated under reduced pressure. The resulting material was purified by silica gel column chromatography using gradient eluent system of hexane:EtOAc (9:1) then hexane:EtOAc:CH_2_Cl_2_ (6.5:1:2.5) leading to compound **4** (40 mg, 0.046 mmol, 63%) as a dark purple solid. ^1^H-NMR (600 MHz, CDCl_3_, 298 K): *δ* 9.80 (s, 1H), 8.93 (d, *J* = 4.1 Hz, 1H), 8.88 (d, *J* = 3.7 Hz, 1H), 7.65 (dd, *J* = 8.4, 1.8 Hz, 1H), 7.60 (d, *J* = 5.9 Hz, 1H), 7.59 (d, *J* = 1.8 Hz, 1H), 7.44 (dd, *J* = 8.5, 2.1 Hz, 1H), 7.36 (dd, *J* = 9.2, 3.1 Hz, 2H), 7.25 (d, *J* = 4.9 Hz, 1H), 6.91 (d, *J* = 8.5 Hz, 1H), 6.87 (d, *J* = 8.6 Hz, 1H), 4.07–4.01 (m, 4H), 3.89 (t, *J* = 7.3 Hz, 2H), 1.92–1.80 (m, 4H), 1.39–1.26 (m, 26H), 0.91–0.85 (m, 15H). ^13^C-NMR (150 MHz, CDCl_3_, 298 K): *δ* 189.8, 161.7, 161.6, 149.8, 148.3, 143.7, 140.1, 139.8, 136.9, 135.1, 131.3, 130.3, 130.2, 129.9, 128.7, 128.41, 128.4, 128.3, 125.4, 124.7, 124.6, 124.15, 123.8, 116.1, 114.9, 108.1, 107.9, 53.4, 48.2, 45.9, 39.2, 39.0, 31.7, 30.4, 30.3, 30.2, 29.2, 29.1, 28.6, 28.3, 26.7, 26.6, 23.6, 23.5, 23.1, 23.0, 22.6, 14.1, 14.0, 14.03, 10.5, 10.51. HRMS (TOF ASAP+, *m/z*): observed 862.4103, calculated for C_51_H_64_N_3_O_3_S_3_ [M + H]^+^ 862.4104.

#### 3.2.3. (*E*)-3-(7-(5-(2,5-Bis(2-ethylhexyl)-3,6-dioxo-4-(thiophen-2-yl)-2,3,5,6-tetrahydropyrrolo-[3,4-c]pyrrol-1-yl)thiophen-2-yl)-10-octyl-10*H*-phenothiazin-3-yl)-2-cyanoacrylic Acid (**D1**)

Compound **4** (47.0 mg, 0.055 mmol, 1 eq.) and cyanoacetic acid (93.0 mg, 1.09 mmol, 20 eq.) were dissolved in acetonitrile (4 mL) up on stirring at 80 °C for a few minutes under nitrogen atmosphere. Piperidine (65 μL, 55.7 mg, 0.654 mmol, 12 eq.) was added and then continue stirring the reaction mixture for 2 h at 80 °C. After cooling to rt. it was quenched by aqueous HCl (2 M, 10 mL). Then wash with water (3 × 20 mL) and the aqueous layer was extracted with CH_2_Cl_2_ (3 × 10 mL). The organic phase was washed using brine (25 mL), then the combined organic extracts were dried over Na_2_SO_4_, filtered and concentrated under reduced pressure. The resulting material was purified by silica gel column chromatography using (gradient: 0–10% MeOH in CH_2_Cl_2_) leading to compound **D1** (45 mg, 0.048 mmol, 89%) as a dark blue solid, mp. 125–130 °C. ^1^H-NMR [600 MHz, DMSO-*d_6_*: CDCl_3_ (4:1 *v/v*), 298 K]: *δ* 8.87 (d, *J* = 4.1 Hz, 1H), 8.78 (d, *J* = 3.7 Hz, 1H), 7.97 (d, *J* = 4.8 Hz, 1H), 7.81 (d, *J* = 7.9 Hz, 1H), 7.73 (s, 1H), 7.65 (d, *J* = 4.1 Hz, 1H), 7.51–7.49 (m, 2H), 7.34–7.33 (m, 1H), 7.09 (d, *J* = 8.6 Hz, 1H), 7.05–7.00 (m, 2H), 3.96–3.93 (m, 4H), 3.53 (br, 2H), 1.51–1.48 (m, 2H), 1.43–2.40 (m, 2H), 1.29–1.24 (m, 26H), 0.87–0.80 (m, 15H). ^13^C-NMR [150 MHz, DMSO-d_6_: CDCl_3_ (4:1 *v/v*), 298 K]: *δ* 174.4, 160.7, 160.5, 148.1, 143.4, 139.2, 138.9, 136.5, 134.4, 133.5, 133.4, 133.1, 132.0, 129.2, 128.2, 127.5, 127.3, 125.3, 124.1, 123.9, 123.3, 122.5, 116.3, 115.6, 111.6, 107.1, 106.9, 69.8, 55.2, 47.0, 45.0, 33.6, 31.3, 31.1, 29.0, 28.9, 28.8, 28.7, 28.6, 28.5, 25.9, 22.4, 22.1, 22.0, 13.9, 13.9, 13.8, 13.7. IR (neat, cm^−1^) ν: 2969 (s), 2931 (w), 2883 (w), 1466 (w), 1378 (s), 1305 (m), 1159 (s), 1127 (s), 1107 (s), 950 (s), 816 (s), 635 (w, br), 487 (w), 424 (w). HRMS (TOF ASAP+, *m/z*): observed 885.4281, calculated for C_53_H_65_N_4_O_2_S_3_ [M–CO_2_ + H]^+^ 885.4270.

### 3.3. Synthesis of Dye **D2**

#### 3.3.1. 7-(5-(2,5-Bis(2-ethylhexyl)-4-(5-(10-hexyl-10*H*-phenothiazin-3-yl)thiophen-2-yl)-3,6-dioxo-2,3,5,6-tetrahydropyrrolo[3,4-c]pyrrol-1-yl)thiophen-2-yl)-10-octyl-10*H*-phenothiazine-3-carbaldehyde (**6**)

In a Schlenk tube compound **4** (40.0 mg, 0.046 mmol, 1.0 eq.), 3-bromo-10-hexyl-10*H*-phenothiazine (**5**, 20.9 mg, 0.058 mmol, 1.2 eq.), Pd_2_(dba)_3_ (3.47 mg, 0.0038 mmol, 8 mol%), (*o*-MeOPh)_3_P (2.67 mg, 0.0076 mmol, 16 mol%), Cs_2_CO_3_ (17.2 mg, 0.053 mmol, 1.1 eq.), pivalic acid (0.98 mg, 0.0096 mmol, 0.2 eq.) were added under nitrogen and then dissolved in dry toluene (1.0 mL). The reaction mixture was stirred for 2 h at 120 °C. After cooling to rt. the reaction mixture was dissolved in CH_2_Cl_2_ and then poured in water. The aqueous layer was extracted with CH_2_Cl_2_ (3 × 15 mL). The combined organic extracts were dried over Na_2_SO_4_, filtered and concentrated under reduced pressure. The resulting material was purified by silica–gel column chromatography using gradient eluent system of hexane:EtOAc (9:1) then hexane:EtOAc:CH_2_Cl_2_ (9:1:1) leading to compound **6** (26 mg, 0.023 mmol, 49%) as a dark blue solid. ^1^H-NMR (600 MHz, CDCl_3_, 298 K): *δ* 9.80 (s, 1H), 8.95 (d, *J* = 4.1 Hz, 1H), 8.91 (d, *J* = 4.1 Hz, 1H), 7.63 (dd, *J* = 8.4, 1.9 Hz, 1H), 7.58 (d, *J* = 1.9 Hz, 1H), 7.43–7.41 (m, 2H), 7.37 (d, *J* = 2.1 Hz, 1H), 7.34 (d, *J* = 2.1 Hz, 1H), 7.32 (dd, *J* = 7.0, 4.1 Hz, 2H), 7.17–7.12 (m, 2H), 6.93 (t, *J* = 7.8 Hz, 1H), 6.89–6.82 (m, 4H), 4.09–4.02 (m, 4H), 3.88–3.83 (m, 4H), 1.93–1.91 (m, 2H), 1.82–1.80 (m, 4H), 1.45–1.27 (m, 32H), 0.92–0.86 (m, 18H). ^13^C-NMR (150 MHz, CDCl_3_, 298 K): *δ* 189.8, 161.7, 161.6, 149.7, 149.0, 147.9, 145.7, 144.4, 143.5, 139.8, 139.2, 137.0, 136.6, 131.3, 130.2, 128.7, 128.5, 128.3, 127.9, 127.4, 127.4, 127.4, 125.5, 125.3, 125.1, 124.6, 124.5, 124.0, 123.8, 123.8, 123.4, 122.7, 116.0, 115.4, 115.4, 114.8, 108.2, 107.9, 53.4, 48.2, 47.6, 46.0, 39.2, 39.2, 31.7, 31.4, 30.3, 29.1, 29.13, 28.6, 26.8, 26.7, 26.6, 26.6, 23.7, 23.1, 22.7, 22.6, 14.1, 14.0, 13.9, 10.6. HRMS (TOF ASAP+, *m/z*): observed 1143.5323, calculated for C_69_H_83_N_4_O_3_S_4_ [M + H]^+^ 1143.5343.

#### 3.3.2. (*E*)-3-(7-(5-(2,5-Bis(2-ethylhexyl)-4-(5-(10-hexyl-10*H*-phenothiazin-3-yl)thiophen-2-yl)-3,6-dioxo-2,3,5,6-tetrahydropyrrolo[3,4-c]pyrrol-1-yl)thiophen-2-yl)-10-octyl-10*H*-phenothiazin-3-yl)-2-cyanoacrylic Acid (**D2**)

Compound **6** (30.0 mg, 0.026 mmol, 1 eq.) and cyanoacetic acid (44.6 mg, 0.525 mmol, 20 eq.) were dissolved in acetonitrile (4 mL) up on stirring at 80 °C for a few minutes under nitrogen atmosphere. Piperidine (31 μL, 26.8 mg, 0.315 mmol, 12 eq.) was added and then continue stirring the reaction mixture for 2 h at 80 °C. After cooling to rt. it was quenched by aqueous HCl (2 M, 10 mL). Then washed with water (3 × 20 mL) and the aqueous layer was extracted with CH_2_Cl_2_ (3 × 10 mL). The organic phase was washed using brine (25 mL), then the combined organic extracts were dried over Na_2_SO_4_, filtered and concentrated under reduced pressure. The resulting material was purified by silica gel column chromatography using (gradient: 0–10% MeOH in CH_2_Cl_2_) leading to compound **D2** (27 mg, 0.022 mmol, 85%) as a dark blue solid. ^1^H-NMR [400 MHz, CDCl_3_: CD_3_OD (4:1 *v/v*), 298 K]: *δ* 8.90 (d, *J* = 3.8 Hz, 1H), 8.87 (d, *J* = 3.7 Hz, 1H), 8.02 (s, 1H), 7.79 (s, 1H), 7.58 (s, 1H), 7.43–7.38 (m, 4H), 7.19–7.12 (m, 3H), 6.96–6.82 (m, 6H), 4.10–3.99 (m, 1H), 3.89–3.79 (m, 4H), 1.96–1.87 (m, 2H), 1.85–1.75 (m, 4H), 1.48–1.91 (m, 36H), 0.93–0.89 (m, 18H). ^13^C-NMR [150 MHz, DMSO-d_6_: CDCl_3_ (4:1 *v/v*), 298 K]: *δ* 173.4, 160.6, 160.5, 147.2, 143.9, 138.2, 136.4, 136.1, 130.3, 128.0, 127.8, 127.3, 126.8, 126.7, 126.5, 124.4, 123.5, 123.2, 123.1, 122.7, 121.9, 115.4, 114.9, 107.6, 107.3, 107.1, 46.7, 44.9, 38.7, 32.9, 31.3, 31.1, 30.8, 29.7, 29.0, 28.9, 28.8, 28.7, 28.6, 28.5, 27.9, 26.1, 25.8, 24.3, 24.2, 24.1, 24.0, 23.9, 23.8, 23.7, 23.5, 22.5, 22.0, 21.9, 13.1, 13.0, 12.9, 9.4. IR (neat, cm^−1^) ν: 2954 (s), 2921 (s), 2869 (w), 2853.96 (w), 1661 (m), 1555 (m), 1465 (s), 1432.41 (s), 1401 (s), 1377 (s), 1250 (w), 1214 (w), 1164 (w), 1087 (w), 1027 (w), 887 (w), 806 (w), 734 (w). HRMS (TOF ASAP+, *m/z*): observed 1209.5327, calculated for C_72_H_83_N_5_O_4_S_4_ [M]^+^ 1209.5328.

### 3.4. Synthesis of Dye **D3**

#### 3.4.1. 4-(5-(2,5-Bis(2-ethylhexyl)-3,6-dioxo-4-(thiophen-2-yl)-2,3,5,6-tetrahydropyrrolo[3,4-c] pyrrol-1-yl)thiophen-2-yl)benzaldehyde (**8**)

In a Schlenk tube compound **2** (47.0 mg, 0.254 mmol, 1.0 eq.), 4-bromobenzaldehyde (**7**, 200 mg, 0.381 mmol, 1.5 eq.), Pd_2_(dba)_3_ (8.24 mg, 0.009 mmol, 3.5 mol%), (*o*-MeOPh)_3_P (6.34 mg, 0.018 mmol, 7 mol%), Cs_2_CO_3_ (91.0 mg, 0.279 mmol, 1.1 eq.), pivalic acid (5.19 mg, 0.051 mmol, 0.2 eq.) were added under nitrogen and then dissolved in dry toluene (2.0 mL). The reaction mixture was stirred for overnight at 120 °C. After cooling to rt. the reaction mixture was dissolved in CH_2_Cl_2_ and then poured in water. The aqueous layer was extracted with CH_2_Cl_2_ (3 × 15 mL). The combined organic extracts were dried over Na_2_SO_4_, filtered and concentrated under reduced pressure. The resulting material was purified by silica gel column chromatography using gradient eluent system of hexane:EtOAc (9:1) then hexane:EtOAc:CH_2_Cl_2_ (9:1:1) leading to compound **8** (102 mg, 0.162 mmol, 64%) as a dark blue solid. ^1^H-NMR (400 MHz, CDCl_3_, 298 K): *δ* 10.03 (s, 1H), 8.94–8.92 (m, 2H), 7.94–7.92 (m, 2H), 7.83 (d, *J* = 8.3 Hz, 2H), 7.65 (dd, *J* = 5.0, 1.1 Hz, 1H), 7.59 (d, *J* = 4.2 Hz, 1H), 7.29–7.27 (m, 1H), 4.08–4.02 (m, 4H), 1.91–1.86 (m, 2H), 1.41–1.23 (m, 16H), 0.93–0.84 (m, 12H). ^13^C-NMR (100 MHz, CDCl_3_, 298 K): *δ* 191.2, 161.7, 161.6, 147.1, 140.8, 139.3, 138.7, 136.4, 135.9, 135.6, 130.9, 130.6, 130.5, 129.7, 128.5, 126.3, 126.1, 108.7, 108.0, 45.9, 39.3, 39.0, 30.3, 30.2, 28.6, 28.3, 23.7, 23.5, 23.1, 23.0, 14.1, 14.0, 10.6, 10.5. HRMS (TOF ASAP+, *m/z*): observed 629.2867, calculated for C_37_H_45_N_2_O_3_S_2_ [M + H]^+^ 629.2866. The ^1^H-NMR data was in accordance with reported values [26].

#### 3.4.2. 4-(5-(2,5-Bis(2-ethylhexyl)-4-(5-(10-hexyl-10*H*-phenothiazin-3-yl)thiophen-2-yl)-3,6-dioxo-2,3,5,6-tetrahydropyrrolo[3,4-c]pyrrol-1-yl)thiophen-2-yl)benzaldehyde (**9**)

In a Schlenk tube compound **8** (70.0 mg, 0.111 mmol, 1.0 eq.), 3-bromo-10-hexyl-10*H*-phenothiazine (**5**, 48.4 mg, 0.134 mmol, 1.2 eq.), Pd_2_(dba)_3_ (4.07 mg, 4.44 µmol, 4.0 mol%), (*o*-MeOPh)_3_P (3.13 mg, 8.88 mmol, 8 mol%), Cs_2_CO_3_ (54.2 mg, 0.166 mmol, 1.5 eq.), pivalic acid (3.06 mg, 0.029 mmol, 0.2 eq.) were added under nitrogen and then dissolved in dry toluene (1.0 mL). The reaction mixture was stirred for overnight at 120 °C. After cooling to rt. the reaction mixture was dissolved in CH_2_Cl_2_ and then poured in water. The aqueous layer was extracted with CH_2_Cl_2_ (3 × 15 mL). The combined organic extracts were dried over Na_2_SO_4_, filtered and concentrated under reduced pressure. The resulting material was purified by silica gel column chromatography using gradient eluent system of hexane:EtOAc (9:1) then hexane:EtOAc:CH_2_Cl_2_ (9:1:1) leading to compound **9** (55 mg, 0.06 mmol, 54%) as a dark blue solid. ^1^H-NMR (600 MHz, CDCl_3_, 298 K): *δ* 10.00 (s, 1H), 9.00 (d, *J* = 4.1 Hz, 1H), 8.89 (d, *J* = 4.1 Hz, 1H), 7.90 (d, *J* = 8.3 Hz, 2H), 7.79 (d, *J* = 8.2 Hz, 2H), 7.55 (d, *J* = 4.1 Hz, 1H), 7.42 (dd, *J* = 8.4, 2.1 Hz, 1H), 7.37 (d, *J* = 2.1 Hz, 1H), 7.32 (d, *J* = 4.1 Hz, 1H), 7.18–7.21 (m, 2H), 6.95–6.92 (m, 1H), 6.84 (dd, *J* = 21.3, 8.3 Hz, 2H), 4.10–4.01 (m, 4H), 3.86–3.83 (m, 2H), 1.94–1.90 (m, 2H), 1.84–1.79 (m, 2H), 1.46–1.24 (m, 22H), 0.93–0.86 (m, 15H). ^13^C-NMR (150 MHz, CDCl_3_, 298 K): *δ* 191.1, 161.7, 161.3, 149.6, 146.7, 145.8, 144.3, 140.8, 138.7, 138.3, 137.6, 136.1, 135.7, 130.8, 130.5, 127.7, 127.5, 127.4, 127.3, 126.2, 126.0, 125.5, 125.2, 124.5, 123.7, 123.5, 122.8, 115.5, 115.4, 109.0, 107.8, 47.6, 46.1, 46.0, 39.3, 39.2, 31.4, 30.3, 28.6, 26.7, 26.6, 23.7, 23.6, 23.2, 23.1, 22.6, 14.1, 13.9, 10.6, 10.5. HRMS (TOF ASAP+, *m/z*): observed 910.4095, calculated for C_55_H_64_N_3_O_3_S_3_ [M + H]^+^ 910.4104.

#### 3.4.3. (*E*)-3-(4-(5-(2,5-Bis(2-ethylhexyl)-4-(5-(10-hexyl-10*H*-phenothiazin-3-yl)thiophen-2-yl)-3,6-dioxo-2,3,5,6-tetrahydropyrrolo[3,4-c]pyrrol-1-yl)thiophen-2-yl)phenyl)-2-cyanoacrylic Acid (**D3**)

Compound **9** (51.0 mg, 0.056 mmol, 1 eq.) and cyanoacetic acid (95 mg, 1.12 mmol, 20 eq.) were dissolved in acetonitrile (4 mL) up on stirring at 80 °C for a few minutes under nitrogen atmosphere. Piperidine (67 μL, 57.2 mg, 0.672 mmol, 12 eq.) was added and then continue stirring the reaction mixture for 1 h at 80 °C. The solubility of the reaction mixture was improved by adding chloroform (1 mL) and continued stirring for another 2 h. After cooling to rt. it was quenched by aqueous HCl (2 M, 10 mL). Then washed with water (3 × 50 mL) and the aqueous layer was extracted with CH_2_Cl_2_ (3 × 15 mL). The organic phase was washed using brine (25 mL), then the combined organic extracts were dried over Na_2_SO_4_, filtered and concentrated under reduced pressure. The resulting material was purified by silica gel column chromatography using (gradient: 0–10% MeOH in CH_2_Cl_2_) leading to compound **D3** (44 mg, 0.045 mmol, 80%) as a dark blue solid, mp. 195–200 °C. ^1^H-NMR [600 MHz, DMSO-d_6_: CDCl_3_ (4:1 *v/v*), 298 K]: *δ* 8.92 (d, *J* = 3.9 Hz, 1H), 8.89 (d, *J* = 3.7 Hz, 1H), 8.03 (s, 1H), 7.92 (d, *J* = 6.3 Hz, 2H), 7.82–7.78 (m, 3H), 7.60 (d, *J* = 3.6 Hz, 1H), 7.46–7.44 (m, 2H), 7.19 (t, *J* = 7.6 Hz, 1H), 7.11 (d, *J* = 7.1 Hz, 1H), 6.95 (d, *J* = 7.6 Hz, 2H), 6.91 (d, *J* = 8.4 Hz, 1H), 3.96–3.88 (m, 4H), 3.83 (t, *J* = 6.6 Hz, 2H), 1.84–1.83 (br, 2H), 1.71–1.69 (m, 2H), 1.39–1.24 (m, 22H), 0.88–0.82 (m, 15H). ^13^C-NMR [150 MHz, DMSO-d_6_: CDCl_3_ (4:1 *v/v*), 298 K]: *δ* 160.7, 160.4, 148.7, 147.3, 145.2, 143.7, 139.3, 137.8, 136.8, 136.0, 134.4, 130.3, 129.1, 127.5, 127.0, 126.6, 125.8, 125.7, 125.1, 124.3, 123.8, 122.6, 122.5, 115.6, 107.7, 106.9, 46.8, 45.2, 30.8, 29.7, 27.9, 26.0, 25.8, 23.2, 22.5, 22.0, 13.9, 13.8, 13.7, 10.2. IR (neat, cm^−1^) ν: 2955 (s), 2923 (s), 2870 (w), 1665 (m), 1591 (w), 1553 (m), 1464 (m), 1432 (w), 1399 (w), 1377 (m), 1284 (w), 1250 (w), 1233 (w), 1189 (w), 1162 (w), 1087 (w), 813 (w), 759 (w), 733 (w), HRMS (TOF ASAP+, *m/z*): observed 933.4259, calculated for C_57_H_65_N_4_O_2_S_3_ [M–CO_2_ + H]^+^ 933.4264.

### 3.5. Synthesis of Dye **D4**

#### 3.5.1. 3-(5-Bromothiophen-2-yl)-2,5-bis(2-ethylhexyl)-6-(thiophen-2-yl)-2,5-dihydropyrrolo [3,4-c]pyrrole-1,4-dione (**10**)

Compound **2** (230 mg, 0.438 mmol, 1.0 eq.) were dissolved in chloroform (15 mL) and NBS (55.0 mg, 0.307 mmol, 1.0 eq.) was added in one batch, and then the reaction mixture was protected from light and stirred at rt. for overnight before water (20 mL) was added and the mixture was extracted with chloroform (3 × 15 mL). The combined organic extracts were dried over Na_2_SO_4_, filtered and concentrated under reduced pressure. The resulting material was purified by silica gel column chromatography using hexane: CH_2_Cl_2_ (1:1) leading to compound **10** (92 mg, 0.152 mmol, 46%) as a dark purple solid. ^1^H-NMR (400 MHz, CDCl_3_, 298 K): *δ* 8.90 (dd, *J* = 3.9, 1.1 Hz, 1H), 8.63 (d, *J* = 4.2 Hz, 1H), 7.64 (dd, *J* = 5.0, 1.1 Hz, 1H), 7.28–7.27 (m, 1H), 7.22 (d, *J* = 4.2 Hz, 1H), 4.03–3.92 (m, 4H), 1.86–1.84 (m, 2H), 1.35–1.17 (m, 16H), 0.91–0.82 (m, 12H). ^13^C-NMR (100 MHz, CDCl_3_, 298 K): *δ* 161.6, 161.5, 140.9, 138.9, 135.5, 135.1, 131.3, 131.2, 130.8, 129.7, 128.5, 118.6, 108.1, 107.8, 45.9, 43.7, 39.1, 39.0, 30.6, 30.1, 28.3, 23.5, 23.5, 23.0, 15.0, 14.0, 10.4. HRMS (TOF ASAP+, *m/z*): observed 603.1709, calculated for C_30_H_40_BrN_2_O_2_S_2_ [M + H]^+^ 603.1709. The ^1^H-NMR data are in good accordance with those previously reported [41].

#### 3.5.2. 3-(5-(4-(Bis(4′-butyl-[1,1′-biphenyl]-4-yl)amino)phenyl)thiophen-2-yl)-2,5-bis (2-ethylhexyl)-6-(thiophen-2-yl)-2,5-dihydropyrrolo[3,4-c]pyrrole-1,4-dione (**12**)

Compound **10** (92.0 mg, 0.152 mmol, 1.0 eq.), compound **11** (126 mg, 0.198 mmol, 1.3 eq.), Pd(PPh_3_)_4_ (17.6 mg, 0.015 mmol, 10 mol%.), and K_2_CO_3_ (0.42 g, 3.04 mmol, 20.0 eq.) were added before the flask was evacuated and nitrogen atmosphere established. Then toluene (7.6 mL), ethanol (1.5 mL), and degassed deionized water (3.04 mL) add and stirred for 24 h at 110 °C. After cooling to rt. the reaction mixture was poured into water (20 mL). The aqueous layer was extracted with CH_2_Cl_2_ (3 × 15 mL). The combined organic extracts were dried over Na_2_SO_4_, filtered and concentrated under reduced pressure. The resulting material was purified by silica gel column chromatography using (gradient: 0–50% CH_2_Cl_2_ in hexane) leading to compound **12** (92.2 mg, 0.091 mmol, 60%) as a dark purple solid. ^1^H-NMR (400 MHz, CDCl_3_, 298 K): *δ* 9.03 (d, *J* = 4.2 Hz, 1H), 8.86 (dd, *J* = 3.9, 1.1 Hz, 1H), 7.60 (dd, *J* = 5.0, 1.1 Hz, 1H), 7.58–7.50 (m, 10H), 7.39 (d, *J* = 4.2 Hz, 1H), 7.27–7.21 (m, 9H), 7.17 (d, *J* = 8.8 Hz, 2H), 4.08–4.03 (m, 4H), 2.67–2.63 (m, 4H), 1.95–1.86 (m, 2H), 1.68–1.63 (m, 4H), 1.44–1.23 (m, 20H), 0.97–0.84 (m, 18H). ^13^C-NMR (100 MHz, CDCl_3_, 298 K): *δ* 161.9, 161.6, 150.1, 148.4, 145.9, 141.9, 140.6, 139.4, 137.7, 137.4, 136.4, 134.9, 130.1, 130.0, 128.9, 128.4, 127.8, 127.7, 127.0, 126.8, 126.6, 125.0, 123.5, 123.1, 108.2, 107.6, 45.94, 45.89, 39.2, 39.1, 35.3, 33.6, 30.3, 30.2, 28.5, 28.3, 23.7, 23.6, 23.10, 23.07, 22.4, 14.08, 14.03, 13.98, 10.58, 10.51. HRMS (TOF ASAP+, *m/z*): observed 1032.5538, calculated for C_68_H_78_N_3_O_2_S_2_ [M + H]^+^ 1032.5530.

#### 3.5.3. 4-(5-(4-(5-(4-(Bis(4′-butyl-[1,1′-biphenyl]-4-yl)amino)phenyl)thiophen-2-yl)-2,5-bis (2-ethylhexyl)-3,6-dioxo-2,3,5,6-tetrahydropyrrolo[3,4-c]pyrrol-1-yl)thiophen-2-yl)benzaldehyde (**13**)

In a Schlenk tube compound **12** (30.0 mg, 0.029 mmol, 1.0 eq.), 4-bromobenzaldehyde (**7**, 6.45 mg, 0.035 mmol, 1.2 eq.), Pd_2_(dba)_3_ (1.07 mg, 1.17 µmol, 4 mol%), (*o*-MeOPh)_3_P (0.825 mg, 2.34 µmol, 8 mol%), Cs_2_CO_3_ (14.0 mg, 42.9 µmol, 1.5 eq.), pivalic acid (0.80 mg, 7.83 µmol, 27%) were added under nitrogen and then dissolved in dry toluene (1.0 mL). The reaction mixture was stirred for overnight at 120 °C. After cooling to rt. the reaction mixture was poured in water (20 mL). The aqueous layer was extracted with CH_2_Cl_2_ (3 ×15 mL). The combined organic extracts were dried over Na_2_SO_4_, filtered and concentrated under reduced pressure. The resulting material was purified by silica gel column chromatography using CH_2_Cl_2_ as eluent leading to compound **13** (37 mg, 0.032 mmol, 67%) as a dark blue solid. ^1^H-NMR (600 MHz, CDCl_3_, 298 K): *δ* 10.02 (s, 1H), 9.07 (d, *J* = 4.1 Hz, 1H), 8.90 (d, *J* = 4.1 Hz, 1H), 7.92 (d, *J* = 8.3 Hz, 2H), 7.81 (d, *J* = 8.3 Hz, 2H), 7.58–7.55 (m, 3H), 7.54–7.50 (m, 8H), 7.39 (d, *J* = 4.1 Hz, 1H), 7.25–7.22 (m, 8H), 7.17 (d, *J* = 8.7 Hz, 2H), 4.12–4.03 (m, 4H), 2.67–2.64 (m, 4H), 1.97–1.91 (m, 2H), 1.67–1.56 (m, 4H), 1.42–1.26 (m, 20H), 0.96–0.85 (m, 18H). ^13^C-NMR (150 MHz, CDCl_3_, 298 K): *δ* 191.2, 161.9, 161.4, 150.6, 148.5, 146.7, 145.9, 141.9, 141.0, 138.8, 138.2, 137.9, 137.7, 136.5, 136.0, 135.8, 130.9, 130.5, 128.8, 127.9, 127.6, 127.0, 126.6, 126.5, 126.2, 126.1, 125.1, 123.6, 123.0, 109.1, 107.7, 46.1, 46.0, 39.3, 39.2, 35.2, 33.6, 30.4, 30.3, 28.6, 28.5, 23.7, 23.1, 22.4, 14.0, 13.9, 10.6. HRMS (TOF ASAP+, *m/z*): observed 1135.5751, calculated for C_75_H_81_N_3_O_3_S_2_ [M]^+^ 1135.5719.

#### 3.5.4. (*E*)-3-(4-(5-(4-(5-(4-(Bis(4′-butyl-[1,1′-biphenyl]-4-yl)amino)phenyl)thiophen-2-yl)-2,5-bis (2-ethylhexyl)-3,6-dioxo-2,3,5,6-tetrahydropyrrolo[3,4-c]pyrrol-1-yl)thiophen-2-yl)phenyl)-2-cyanoacrylic Acid (**D4**)

Compound **16** (34.0 mg, 0.030 mmol, 1 eq.) and cyanoacetic acid (50.9 mg, 0.598 mmol, 20 eq.) were dissolved in acetonitrile (4 mL) up on stirring at 80 °C for a few minutes under nitrogen atmosphere. Piperidine (36 μL, 30.6 mg, 0.359 mmol, 12 eq.) was added and the reaction mixture further stirred at 80 °C for 1 h. The solubility of the reaction mixture was improved by adding chloroform (1 mL) and continued the stirring for another 2 h. After cooling to rt., the reaction mixture was quenched by aqueous HCl (2 M, 10 mL) and washed with water (3 × 50 mL). The collected aqueous layer was extracted with CH_2_Cl_2_ (3 × 15 mL). The organic phase was washed using brine (25 mL), then the combined organic extracts were dried over Na_2_SO_4_, filtered and concentrated under reduced pressure. The resulting material was purified by silica gel column chromatography using (gradient: 0–10% MeOH in CH_2_Cl_2_) leading to compound **D4** (35 mg, 0.029 mmol, 97%) as a dark blue solid. ^1^H-NMR [600 MHz, DMSO-d_6_: CDCl_3_ (4:1 *v/v*), 298 K]: *δ* 8.99 (d, *J* = 3.7 Hz, 1H), 8.92 (d, *J* = 3.5 Hz, 1H), 7.97 (s, 1H), 7.84 (s, 2H), 7.80 (s, 1H), 7.72 (d, *J* = 6.3 Hz, 2H), 7.59–7.54 (m, 7H), 7.49 (d, *J* = 7.9 Hz, 4H), 7.21 (d, *J* = 7.9 Hz, 4H), 7.11 (d, *J* = 8.2 Hz, 4H), 7.04 (d, *J* = 8.2 Hz, 2H), 3.90 (br, 4H), 2.62–2.61 (m, 4H), 1.84 (br, 2H), 1.61–1.56 (m, 4H), 1.36–1.24 (m, 20H), 0.94–0.81 (m, 18H). ^13^C-NMR [150 MHz, DMSO-d_6_: CDCl_3_ (4:1 *v/v*), 298 K]: *δ* 160.6, 160.4, 149.6, 147.6, 147.2, 145.3, 141.2, 139.3, 137.7, 136.8, 136.0, 135.5, 130.3, 129.1, 128.7, 127.4, 126.9, 126.8, 126.0, 125.6, 124.8, 122.3, 107.6, 106.8, 34.5, 33.0, 29.0, 28.0, 27.9, 23.1, 22.5, 22.1, 21.8, 13.8, 13.7, 10.2. IR (neat, cm^−1^) ν: 2954 (s), 2920 (s), 2869 (w), 1666 (s), 1595 (s), 1553 (s), 1494 (s), 1457 (s), 1434 (w), 1399 (w), 1377 (s), 1322 (m), 1293 (w), 1234 (w), 1188 (w), 813 (m), 732 (w). HRMS (ESI+, *m/z*): observed 1203.5861, calculated for C_78_H_83_N_4_O_4_S_2_ [M + H]^+^ 1203.5856.

### 3.6. Synthesis of Dye **D5**

#### 3.6.1. 7-(5-(4-(5-(4-(Bis(4′-butyl-[1,1′-biphenyl]-4-yl)amino)phenyl)thiophen-2-yl)-2,5-bis (2-ethylhexyl)-3,6-dioxo-2,3,5,6-tetrahydropyrrolo[3,4-c]pyrrol-1-yl)thiophen-2-yl)-10-octyl-10*H*-phenothiazine-3-carbaldehyde (**15**)

In a Schlenk tube compound **12** (42.0 mg, 0.041 mmol, 1.0 eq.), **3** (20.4 mg, 0.049 mmol, 1.2 eq.), Pd_2_(dba)_3_ (1.50 mg, 1.64 µmol, 4 mol%), (*o*-MeOPh)_3_P (0.156 mg, 3.28 µmol, 8 mol%), Cs_2_CO_3_ (19.7 mg, 60.5 µmol, 1.5 eq.), pivalic acid (1.12 mg, 11.0 µmol, 27%) were added under nitrogen and then dissolved in dry toluene (1.0 mL). The reaction mixture was stirred overnight at 120 °C. After cooling to rt. the reaction mixture was poured in water (20 mL). The aqueous layer was extracted with CH_2_Cl_2_ (3 × 15 mL). The combined organic extracts were dried over Na_2_SO_4_, filtered and concentrated under reduced pressure. The resulting material was purified by silica gel column chromatography using CH_2_Cl_2_ leading to compound **15** (43 mg, 0.031 mmol, 77%) as a dark blue solid. ^1^H-NMR (600 MHz, CDCl_3_, 298 K): *δ* 9.80 (s, 1H), 9.02 (d, *J* = 4.1 Hz, 1H), 8.91 (d, *J* = 4.1 Hz, 1H), 7.64 (dd, *J* = 8.4, 1.9 Hz, 1H), 7.59 (d, *J* = 1.9 Hz, 1H), 7.56–7.50 (m, 10H), 7.44 (dd, *J* = 8.5, 2.1 Hz, 1H), 7.37 (dd, *J* = 7.2, 3.1 Hz, 2H), 7.34 (d, *J* = 4.1 Hz, 1H), 7.25–7.21 (m, 8H), 7.16 (d, *J* = 8.7 Hz, 2H), 6.90 (d, *J* = 8.5 Hz, 1H), 6.87 (d, *J* = 8.6 Hz, 1H), 4.10–4.02 (m, 4H), 3.89 (t, *J* = 7.3 Hz, 2H), 2.66–2.64 (m, 4H), 1.95–1.92 (m, 2H), 1.85–1.80 (m, 2H), 1.67–1.61 (m, 4H), 1.42–1.26 (m, 30H), 0.96–0.86 (m, 21H). ^13^C-NMR (150 MHz, CDCl_3_, 298 K): *δ* 189.8, 161.7, 161.6, 149.9, 149.8, 148.3, 147.8, 145.9, 143.6, 141.8, 140.0, 139.1, 137.7, 137.3, 136.5, 136.4, 131.3, 130.2, 128.9, 128.8, 128.5, 128.4, 127.8, 127.8, 127.0, 126.8, 126.5, 125.3, 125.0, 124.6, 124.1, 123.8, 123.5, 123.1, 116.1, 114.9, 108.2, 107.8, 48.2, 45.9, 39.26, 39.2, 35.2, 33.6, 31.7, 30.4, 30.3, 29.2, 29.1, 28.6, 28.5, 26.7, 26.67, 23.7, 23.1, 23.1, 22.6, 22.4, 14.1, 14.0, 13.9, 10.6. HRMS (TOF ASAP+, *m/z*): observed 1369.6982, calculated for C_89_H_101_N_4_O_3_S_3_ [M + H]^+^ 1369.7030.

#### 3.6.2. (*E*)-3-(7-(5-(4-(5-(4-(Bis(4′-butyl-[1,1′-biphenyl]-4-yl)amino)phenyl)thiophen-2-yl)-2,5 -bis(2-ethylhexyl)-3,6-dioxo-2,3,5,6-tetrahydropyrrolo[3,4-c]pyrrol-1-yl)thiophen-2-yl)-10-octyl-10H-phenothiazin-3-yl)-2-cyanoacrylic Acid (**D5**)

Compound **15** (35.6 mg, 0.026 mmol, 1 eq.) and cyanoacetic acid (44.2 mg, 0.520 mmol, 20 eq.) were dissolved in acetonitrile (4 mL) up on stirring at 80 °C for a few minutes under nitrogen atmosphere. Piperidine (31 μL, 26.6 mg, 0.312 mmol, 12 eq.) was added and then continue stirring the reaction mixture for 1 h at 80 °C. The solubility of the reaction mixture was improved by adding chloroform (1 mL) and continue stirring for another 2 h. After cooling to rt. it was quenched by aqueous HCl (2 M, 10 mL). Then wash with water (3 × 50 mL) and the aqueous layer was extracted with CH_2_Cl_2_ (3 × 15 mL). The organic phase was washed using brine (25 mL), then the combined organic extracts were dried over Na_2_SO_4_, filtered and concentrated under reduced pressure. The resulting material was purified by silica gel column chromatography using (gradient: 0–10% MeOH in CH_2_Cl_2_) leading to compound **D5** (30 mg, 0.020 mmol, 80%) as a dark blue solid, mp. 240–245 °C. ^1^H-NMR [600 MHz, DMSO-d_6_: CDCl_3_ (4:1 *v/v*), 298 K]: *δ* 8.93 (d, *J* = 3.2 Hz, 1H), 8.89 (d, *J* = 3.1 Hz, 1H), 8.01 (s, 1H), 7.78 (d, *J* = 5.9 Hz, 1H), 7.69 (s, 1H), 7.55–7.48 (m, 12H), 7.43–7.39 (m, 2H), 7.21 (d, *J* = 7.9 Hz, 4H), 7.10 (d, *J* = 8.2 Hz, 4H), 7.01–6.98 (m, 3H), 6.91 (d, *J* = 7.7 Hz, 1H), 3.87–3.82 (m, 6H), 2.60 (t, *J* = 7.5 Hz, 4H), 1.82 (br, 2H), 1.68 (br, 2H), 1.60–1.55 (m, 4H), 1.36–1.24 (m, 30H), 0.93–0.79 (m, 21H). ^13^C-NMR [150 MHz, DMSO-d_6_: CDCl_3_ (4:1 *v/v*), 298 K]: *δ* 160.6, 160.5, 149.1, 147.5, 145.3, 142.9, 141.2, 138.6, 138.2, 136.8, 136.4, 135.5, 128.6, 127.6, 127.5, 127.4, 127.2, 127.1, 126.9, 126.6, 126.5, 126.0, 124.8, 123.8, 123.6, 123.5, 123.0, 122.4, 122.2, 107.1, 106.9, 40.2, 40.0, 39.9, 39.8, 39.6, 39.5, 39.3, 39.2, 34.6, 33.1, 31.2, 28.7, 28.6, 26.1, 23.1, 22.7, 22.6, 22.1, 21.8, 13.9, 13.8, 13.7, 10.3, 10.2. IR (neat, cm^−1^) ν: 2954 (s), 2920 (s), 2869 (w), 2854 (w), 1662 (m), 1596 (w), 1555 (m), 1494 (s), 1458 (s), 1433 (s), 1402 (w), 1377 (s), 1322 (m), 1214 (w), 1180 (w), 1087 (w), 1026 (w), 887 (w), 813 (m), 733 (w). HRMS (ESI+, *m/z*): observed 1436.7032, calculated for C_92_H_102_N_5_O_4_S_3_ [M + H]^+^ 1436.7088.

### 3.7. DSSC Fabrication

The photoanodes were fabricated from FTO glass (NSG10, Nippon Sheet Glass, Tokyo, Japan), washed with Deconex 21 (2 g/L) in an ultrasonic bath for 45 min, then cleaned in a UV/Ozone cleaner (PSD PRO-UV T6, Novascan, Chicago, IL, USA) for 15 min. A dense TiO_2_ blocking layer was deposited by hydrothermal deposition of aqueous TiCl_4_ (40 mM in deionized water) at 70 °C for 2 × 45 min, followed by rinsing with deionized water and ethanol. Two active layers (18NR-T, Dyesol, Elanora, Australia) and a scattering layer of TiO_2_ (WER2-O, Dyesol) were screen-printed (54T mesh, 0.283 cm^2^ active area) onto the FTO glass slides. After each layer was printed, the glass slides were heated at a hotplate for 5 min at 125 °C. The photoanodes were sintered in a programmable furnace at 125, 250, 375, 450 and 500 °C for 5, 5, 5, 15 and 15 min, with a ramp time of 10 min between each step. Afterwards, a TiCl_4_ post-treatment was performed following the same procedure as for the blocking layer. Before immersion in the dye staining solutions, the photoanodes were annealed by heat gun at 450 °C for 30 min and allowed to reach approximately 80 °C before immersion. Counter electrodes were fabricated with TEC10 FTO glass (Sigma-Aldrich, St. Louis, MO, USA). Electrolyte filling holes were drilled with a diamond drill bit, and the electrodes rinsed in Deconex 21 (2 g/L) solution, deionized water, ethanol and acetone, each for 15 min under sonication. A thin catalytic layer of Pt was deposited by drop casting a solution of H_2_PtCl_6_ (10 mM in isopropanol, 5 µL/cm^2^), followed by heating at 400 °C for 15 min. The solvent mixture for the staining solutions was tetrahydrofuran/acetonitrile (57:43, *v/v*), dye concentration was 0.5 mM and concentrations of CDCA was either 0 or 5 mM. The photoanodes were stained for 22 h in an oven holding 30 °C and rinsed with acetonitrile before assembly. A Surlyn gasket (25 µm, Solaronix, Aubonne, Swiss) was melted between the photoanode and counter electrodes, and the A6141 electrolyte was injected by vacuum backfilling and the hole sealed with Surlyn and a glass cover. The A6141 electrolyte consisted of 0.60 M 1-butyl-3-methylimidazolium iodide, 0.03 M I_2_, 0.10 M guanidinium thiocyanate and 0.50 M *tert*-butylpyridine in a mixture of acetonitrile and valeronitrile (85:15, *v/v*). The conductive edges of the working and counter electrodes protruding from the device were covered by silver conducting paint (Electrolube, SCP, Ashby Park, UK).

### 3.8. Device Characterization

Current-density-voltage characteristics of the devices were measured under 1 sun AM1.5G illumination by a SP300B solar simulator (Sciencetech, London, UK) equipped with a model 2450 what? (Keithley, Cleveland, OH, USA). The potential scan direction was from short-circuit to open-circuit, and the devices were masked off by a 0.158 cm^2^ black metal mask. Incident photon-to-current conversion efficiency (IPCE) measurements were recorded using a halogen lamp (HL-2000, Ocean-Optics, Dunedin, FL, USA) with a monochromator (CM110 Spectral Products, Putnam, CT, USA) and a Keithley 2450 what? The devices as well as the reference photodiode (FDS100-CAL, Thorlabs, Newton, NJ, USA) were covered with a mask size of 0.049 cm^2^.

### 3.9. Electrochemistry

Cyclic voltammetry was performed using a standard three-electrode cell, glassy carbon as working, silver/silver chloride (Ag/AgCl) as a reference and platinum wire were used as counter electrode. All measurements were performed under N_2_ bubbling into the electrochemical cell for 10 min prior to the measurements. The CV curves were collected using 50 mV/s scan rate from tetra-n-butylammonium hexafluorophosphate (TBAPF_6_, 98%) as the supporting electrolyte in anhydrous acetonitrile. Measurements were recorded using a Model Verstastat 3 potentiostat/galvanostat (EG&G Princeton Applied Research, Gaithersburg, MD, USA). All results were calibrated using commercially available ferrocene as internal standard.

## 4. Conclusions

Diketopyrrolopyrroles are an interesting dye class as they have a broad and intense spectral absorption in the visible and NIR region. Such dyes have until now been synthesized by conventional cross-coupling methodologies. Herein, we show that 3,6-dithienyl–DPP dyes with phenothiazine and triarylamine as donor and phenothiazine and phenyl as π-linker can be synthesized in moderate to good yield using direct C-H arylation. This methodology provides a shorter, more economical and greener route to the synthesis of DPP based dyes. Thus, direct C-H arylation is an important complement to conventional cross-coupling methods, which gives access to alternative synthetic routes and might aid future commercialization of technologies based on organic dyes. The synthesized dyes displayed a broad absorption in the range of 350 to 800 nm with high molar extinction coefficients. DSSC devices fabricated with these dyes exhibited PCE from 2.9 to 3.4%. Although the performance of these dyes can be improved by device engineering, these results alongside previous reports indicate that 3,6-dithienyl–DPP is suboptimal as a core for DPP based dyes for DSSC.

## Figures and Tables

**Table 1:** Photophysical and electrochemical properties of the dyes **D1**–**D5**.

Dye	λ_max_ (nm) ^a^	ε (10^4^ M^−1^ cm^−1^) ^a^	λ_max_ TiO_2_ (nm) ^b^	λ_int._ (nm) ^c^	E_0−0,_ (eV) ^d^	E_HOMO_ (V) ^e^	E_LUMO_ (V) ^f^
**D1**	312, 576	2.995, 3.589	350, 565	611	2.03	1.12	−0.91
**D2**	331, 604	3.020, 4.643	350, 599	649	1.91	1.02	−0.89
**D3**	331, 378, 604	3.390, 3.620, 7.548	381, 578	667	1.86	0.98	−0.88
**D4**	356, 606	4.465, 5.603	380, 595	664	1.86	1.04	−0.82
**D5**	341, 608	5.560, 5.687	597	662	1.87	1.02	−0.85

^a^ Adsorption maxima, measured in CH_2_Cl_2_ solution; ^b^ Adsorption maxima on TiO_2_; ^c^ Intersection of the absorption and normalized emission spectra; ^d^ Calculated from the intersection of the absorption and normalized emission spectra (E_0−0_ = 1240/λ_int_) vs. NHE; ^e^ Determined by cyclic voltammetry in solution vs. NHE; ^f^ E_LUMO_ = E_HOMO_ − E_0−0_.

**Table 2:** Photovoltaic performance of dyes **D1**–**D5** compared with N**719** under 1 sun AM 1.5 G illumination.

Dye	Donor	π-Linker	J_sc_ (mAcm^−2^)	Voc (V)	FF (%)	PCE (%)
**D1 ^a^**	Thiophene	Phenoth.	5.97 ± 0.05	0.67 ± 0.00	73.3 ± 0.00	2.94 ± 0.03
**D2 ^a^**	Phenoth.	Phenoth.	5.62 ± 0.68	0.70 ± 0.00	72.6 ± 0.03	2.83 ± 0.22
**D3 ^a^**	Phenoth.	Phenyl	7.55 ± 0.73	0.63 ± 0.00	61.5 ± 0.00	2.94 ± 0.26
**D4 ^a^**	Triaryl	Phenyl	6.15 ± 0.67	0.63 ± 0.00	69.8 ± 0.02	2.72 ± 0.37
**D5 ^a^**	Triaryl	Phenoth.	6.97 ± 0.21	0.69 ± 0.01	65.9 ± 0.01	3.16 ± 0.02
**D3 ^b^**	Phenoth.	Phenyl	5.24 ± 0.30	0.63 ± 0.00	68.2 ± 0.05	2.27 ± 0.29
**D3 ^c^**	Phenoth.	Phenyl	7.46 ± 0.35	0.63 ± 0.00	71.4 ± 0.01	3.35 ± 0.09
**N719 ^d^**			13.4 ± 0.83	0.71 ± 0.01	68.3 ± 0.03	6.48 ± 0.04

^a^ Staining: THF:acetonitrile (57:43) + CDCA; ^b^ Staining: THF, no CDCA; ^c^ Staining: THF + CDCA; ^d^ Staining: Ethanol + CDCA.

## Supplementary Materials

The following are available online, Synthesis of **11** and **14**; DFT calculations Table S1; Absorption and emission spectra: Figure S1; cyclic voltammogram of dyes: Figure S2. ^13^C and ^1^H-NMR spectra: Figures S3–S13.

## References

[1] Hagfeldt A., Boschloo G., Sun L., Kloo L., Pettersson H. (2010). Dye-sensitized solar cells. Chem. Rev..

[2] Lee C.P., Lin R.Y.-Y., Lin L.Y., Li C.T., Chu T.C., Sun S.S., Lin J.T., Ho K.C. (2015). Recent progress in organic sensitizers for dye-sensitized solar cells. RSC Adv..

[3] Mishra A., Fischer M.K.R., Bauerle P. (2009). Metal-free organic dyes for dye-sensitized solar cells: From structure: Property relationships to design rules. Angew. Chem. Int. Ed. Engl..

[4] Miyaura N., Yamada K., Suzuki A. (1979). A new stereospecific cross-coupling by the palladium-catalyzed reaction of 1-alkenylboranes with 1-alkenyl or 1-alkynyl halides. Tetrahedron Lett..

[5] Suzuki A. (2005). Carbon-carbon bonding made easy. Chem. Commun..

[6] Cordovilla C., Bartolomé C., Martínez-Ilarduya J.M., Espinet P. (2015). The Stille Reaction, 38 Years Later. ACS Catal..

[7] Milstein D., Stille J.K. (1978). A general, selective, and facile method for ketone synthesis from acid chlorides and organotin compounds catalyzed by palladium. J. Am. Chem. Soc..

[8] Milstein D., Stille J.K. (1979). Mechanism of reductive elimination. Reaction of alkylpalladium(II) complexes with tetraorganotin, organolithium, and Grignard reagents. Evidence for palladium(IV) intermediacy. J. Am. Chem. Soc..

[9] Bohra H., Wang M. (2017). Direct C-H arylation: A “Greener” approach towards facile synthesis of organic semiconducting molecules and polymers. J. Mater. Chem. A.

[10] Efrem A., Wang K., Wang M. (2017). Facile synthesis of a narrow-bandgap strong-donor-alt-strong-acceptor copolymer of poly(5,6-difluorobenzo-[c][1,2,5]-thiadiazole-alt-5H-dithieno[3,2-b:2′,3′-d]pyran) via direct C-H arylation polymerization. Dyes Pigment..

[11] Mercier L.G., Leclerc M. (2013). Direct (hetero)arylation: A new tool for polymer chemists. Acc. Chem. Res..

[12] Zhang J., Chen W., Rojas A.J., Jucov E.V., Timofeeva T.V., Parker T.C., Barlow S., Marder S.R. (2013). Controllable direct arylation: Fast route to symmetrical and unsymmetrical 4,7-diaryl-5,6-difluoro-2,1,3-benzothiadiazole derivatives for organic optoelectronic materials. J. Am. Chem. Soc..

[13] Zheng L., Cao Q., Wang J., Chai Z., Cai G., Ma Z., Han H., Li Q., Li Z., Chen H. (2017). Novel D-A-π-A-type organic dyes containing a ladderlike dithienocyclopentacarbazole donor for effective dye-sensitized solar cells. ACS Omega.

[14] Kang X., Zhang J., O’Neil D., Rojas A.J., Chen W., Szymanski P., Marder S.R., El-Sayed M.A. (2014). Effect of molecular structure perturbations on the performance of the D-A-π-A dye sensitized solar cells. Chem. Mater..

[15] Lin P.-H., Lu T.-J., Cai D.-J., Lee K.-M., Liu C.-Y. (2015). Connecting direct C-H arylation reactions with dye-sensitized solar cells: A shortcut to D-A-π-A organic dyes. ChemSusChem.

[16] Huang J.-H., Lin P.-H., Li W.-M., Lee K.-M., Liu C.-Y. (2017). Sn- and Pd-free synthesis of D-π-A organic sensitizers for dye-sensitized solar cells by Cu-catalyzed direct arylation. ChemSusChem.

[17] Sil M.C., Sudhakar V., Mele Kavungathodi M.F., Punitharasu V., Nithyanandhan J. (2017). Orthogonally functionalized donor/acceptor homo- and heterodimeric dyes for dye-sensitized solar cells: An approach to introduce panchromaticity and control the charge recombination. ACS Appl. Mater. Interfaces.

[18] Hao Y., Saygili Y., Cong J., Eriksson A., Yang W., Zhang J., Polanski E., Nonomura K., Zakeeruddin S.M., Graetzel M. (2016). Novel blue organic dye for dye-sensitized solar cells achieving high efficiency in cobalt-based electrolytes and by co-sensitization. ACS Appl. Mater. Interfaces.

[19] Li X., Zheng Z., Jiang W., Wu W., Wang Z., Tian H. (2015). New D-A-π-A organic sensitizers for efficient dye-sensitized solar cells. Chem. Commun..

[20] Qu S., Wu W., Hua J., Kong C., Long Y., Tian H. (2010). New diketopyrrolopyrrole (DPP) dyes for efficient dye-sensitized solar cells. J. Phys. Chem. C.

[21] Singh S.P., Roy M.S., Thomas A., Bhanuprakash K., Sharma G.D. (2012). Effect of linker used in D-A-π-A metal free dyes with different π-spacers for dye sensitized solar cells. Org. Electron..

[22] Duvva N., Raptis D., Kumar C.V., Koukaras E.N., Giribabu L., Lianos P. (2016). Design of diketopyrrolopyrrole chromophores applicable as sensitizers in dye-sensitized photovoltaic windows for greenhouses. Dyes Pigment..

[23] Sun X., Wang Y., Li X., Agren H., Zhu W., Tian H., Xie Y. (2014). Cosensitizers for simultaneous filling up of both absorption valleys of porphyrins: A novel approach for developing efficient panchromatic dye-sensitized solar cells. Chem. Commun..

[24] Tian G., Cai S., Li X., Agren H., Wang Q., Huang J., Su J. (2015). A new D-A-π-A type organic sensitizer based on substituted dihydroindolo [2,3-b] carbazole and DPP unit with a bulky branched alkyl chain for highly efficient DSCs. J. Mater. Chem. A.

[25] Zang X.-F., Huang Z.-S., Wu H.-L., Iqbal Z., Wang L., Meier H., Cao D. (2014). Molecular design of the diketopyrrolopyrrole-based dyes with varied donor units for efficient dye-sensitized solar cells. J. Power Sources.

[26] Holcombe T.W., Yum J.-H., Yoon J., Gao P., Marszalek M., Censo D.D., Rakstys K., Nazeeruddin M.K., Graetzel M. (2012). A structural study of DPP-based sensitizers for DSC applications. Chem. Commun..

[27] Punzi A., Zappimbulso N., Farinola G.M. (2019). Synthesis of novel diketopyrrolopyrrole-based dyes. Mon. Chem..

[28] Yum J.-H., Holcombe T.W., Kim Y., Rakstys K., Moehl T., Teuscher J., Delcamp J.H., Nazeeruddin M.K., Gratzel M. (2013). Blue-coloured highly efficient dye-sensitized solar cells by implementing the diketopyrrolopyrrole chromophore. Sci. Rep..

[29] Yum J.-H., Holcombe T.W., Kim Y., Yoon J., Rakstys K., Nazeeruddin M.K., Grätzel M. (2012). Towards high-performance DPP-based sensitizers for DSC applications. Chem. Commun..

[30] Aouine Y., Alami A., El Hallaoui A., Elachqar A., Zouihri H. (2010). 10-(Prop-2-ynyl)-10H-phenothiazine. Acta Crystallogr. Sect. E Struct. Rep. Online.

[31] McDowell J.J.H. (1976). The crystal and molecular structure of phenothiazine. Acta Crystallogr. Sect. B.

[32] Frebort S., Elias Z., Lycka A., Lunak S., Vynuchal J., Kubac L., Hrdina R., Burgert L. (2011). O- and N-alkylated diketopyrrolopyrrole derivatives. Tetrahedron Lett..

[33] Zhao B., Sun K., Xue F., Ouyang J. (2012). Isomers of dialkyl diketo-pyrrolo-pyrrole: Electron-deficient units for organic semiconductors. Org. Electron..

[34] Nazeeruddin M.K., Pechy P., Renouard T., Zakeeruddin S.M., Humphry-Baker R., Comte P., Liska P., Cevey L., Costa E., Shklover V. (2001). Engineering of efficient panchromatic sensitizers for nanocrystalline TiO_2_-based solar cells. J. Am. Chem. Soc..

[35] Zhang L., Cole J.M. (2017). Dye aggregation in dye-sensitized solar cells. J. Mater. Chem. A.

[36] Espinoza E.M., Clark J.A., Soliman J., Derr J.B., Morales M., Vullev V.I. (2019). Practical aspects of cyclic voltammetry: How to estimate reduction potentials when irreversibility prevails. J. Electrochem. Soc..

[37] Huang J.-H., Jiang K.-J., Zhang F., Wu W., Li S.-G., Yang L.-M., Song Y.-L. (2014). Engineering diketopyrrolopyrrole sensitizers for highly efficient dye-sensitized solar cells: Enhanced light harvesting and intramolecular charge transfer. RSC Adv..

[38] Buene A.F., Ose E.E., Zakariassen A.G., Hagfeldt A., Hoff B.H. (2019). Auxiliary donors for phenothiazine sensitizers for dye-sensitized solar cells—How important are they really?. J. Mater. Chem. A.

[39] Wang W., Li X., Lan J., Wu D., Wang R., You J. (2018). Construction of 3,7-Dithienyl Phenothiazine-Based Organic Dyes via Multistep Direct C-H Arylation Reactions. J. Org. Chem..

[40] Huo L., Hou J., Chen H.-Y., Zhang S., Jiang Y., Chen T.L., Yang Y. (2009). Bandgap and Molecular Level Control of the Low-Bandgap Polymers Based on 3,6-Dithiophen-2-yl-2,5-dihydropyrrolo[3,4-c] pyrrole-1,4-dione toward Highly Efficient Polymer Solar Cells. Macromolecules.

[41] Harschneck T., Zhou N., Manley E.F., Lou S.J., Yu X., Butler M.R., Timalsina A., Turrisi R., Ratner M.A., Chen L.X. (2014). Substantial photovoltaic response and morphology tuning in benzo[1,2-b:6,5-b′]dithiophene (bBDT) molecular donors. Chem. Commun..

