# Supplementary material for: Synthesis of Novel 3,6-Dithienyl Diketopyrrolopyrrole Dyes by Direct C-H Arylation
Source: Molecules. 2020 May 18;25(10):2349. doi: 10.3390/molecules25102349 (PMC7288183; doi:10.3390/molecules25102349)
Supplement: Supplementary file 1 [file molecules-25-02349-s001.pdf]

Supplementary Materials:

Synthesis of novel 3,6-dithienyl diketopyrrolopyrrole dyes by direct C–H arylation

Amsalu Efrem Yemene, Vishwesh Venkatraman, David Moe Almenningen, Bård Helge Hoff*, Odd Reidar Gautun*

Department of Chemistry, Norwegian University of Science and Technology (NTNU), N-7491 Trondheim, Norway

Corresponding author and requests for materials should be addressed to B.H.H and O.R.G (email: bard.h.hoff@ntnu.no and odd.r.gautun@ntnu.no)

Contents

Synthesis of building blocks 11 and 14 2

DFT calculations 4

Absorption and emission spectra of the dyes D1–D5 5

Cyclic voltammogram of dyes D1–D5 6

^{1}H and ^{13}C NMR spectra of key building blocks and dyes 7

Synthesis of building blocks 11 and 14

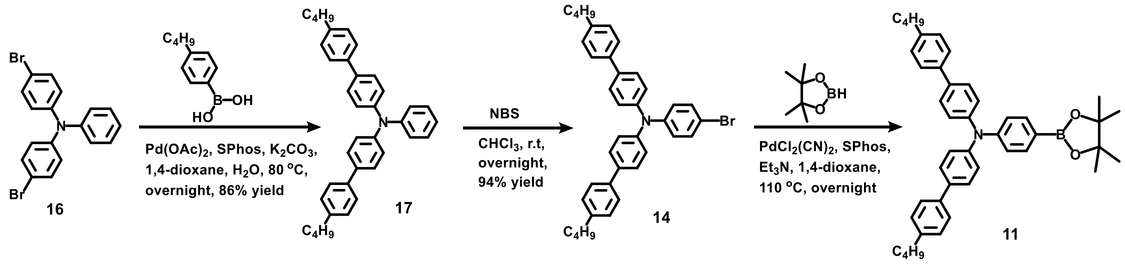

Scheme S1: Synthesis route to **11**.

4'-Butyl-*N*-(4'-butyl-[1,1'-biphenyl]-4-yl)-*N*-phenyl-[1,1'-biphenyl]-4-amine (17). Compound **16** (500 mg, 1.24 mmol, 1.0 eq.), (4-butylphenyl)boronic acid (550 mg, 3.10 mmol, 2.5 eq.), $Pd(OAc)_2$ (11.0 mg, 0.050 mmol, 4 mol%.), SPhos (41.0 mg, 0.099 mmol, 8 mol%), and K_2CO_3 (0.68 g, 4.96 mmol, 4.0 eq.) were added before the flask was evacuated and nitrogen atmosphere established. Then 1,4-dioxane (4.5 mL) and degassed deionized water (4.5 mL) add and stirred for overnight at 80 °C. After cooling to r.t the reaction mixture was quenched by aqueous NaOH (1M, 10 mL). The aqueous layer was extracted with DCM (3x15 mL). The combined organic extracts were dried over Na_2SO_4, filtered and concentrated under reduced pressure. The resulting material was purified by silica gel column chromatography using (gradient: 0–50% DCM in hexane) leading to compound **17** (550 mg, 1.08 mmol, 86%) as a colorless sticky oil. ^1H NMR (400 MHz, $CDCl_3$, 298 K): δ 7.50–7.47 (m, 8H), 7.30–7.22 (m, 6H), 7.18–7.15 (m, 6H), 7.04 (t, J = 7.3 Hz, 1H), 2.66–2.62 (m, 4H), 1.67–1.59 (m, 4H), 1.43–1.34 (m, 4H), 0.98 (t, J = 7.3 Hz, 6H). ^{13}C NMR (100 MHz, $CDCl_3$, 298 K): δ 147.6, 146.7, 141.6, 137.9, 135.3, 129.2, 128.8, 127.6, 126.5, 124.4, 124.1, 122.9, 35.2, 33.6, 22.4, 13.9. HRMS (TOF ASAP+, *m/z*): observed 510.3160, calculated for $C_{38}H_{40}N$ $[M+H]^+$ 510.3155.

***N*-(4-Bromophenyl)-4'-butyl-*N*-(4'-butyl-[1,1'-biphenyl]-4-yl)-[1,1'-biphenyl]-4-amine (14)**. Compound **17** (100 mg, 0.196 mmol, 1.0 eq.) was dissolved in chloroform (10 mL) and NBS (34.9 mg, 0.196 mmol, 1.0 eq.) was added in one batch at r.t. After stirring overnight the reaction mixture was concentrated in vacuo then loaded directly on silica gel column chromatography eluted using DCM:hexane (1:3) leading to compound **14** (108 mg, 0.183 mmol, 94%) as a colorless sticky oil. ^1H NMR (600 MHz, $CDCl_3$, 298 K): δ 7.49–7.47 (m, 8H), 7.36 (d, J = 8.7 Hz, 1H), 7.29–7.22 (m, 6H), 7.18–7.14 (m, 4H), 7.03 (d, J = 8.8 Hz, 1H), 2.64 (t, J = 7.7 Hz, 4H), 1.66–1.61 (m, 4H), 1.42–1.36 (m, 4H), 0.94 (t, J = 7.4 Hz, 6H). ^{13}C NMR

(150 MHz, $CDCl_3$, 298 K): δ 147.64, 146.74, 141.6, 138.0, 136.0, 129.3, 128.8, 127.64, 126.5, 124.5, 124.2, 123.0, 35.3, 33.7, 22.4, 14.0. HRMS (TOF ASAP+, *m/z*): observed 588.2271, calculated for $C_{38}H_{39}BrN$ $[M+H]^+$ 588.2260.

4'-Butyl-*N*-(4'-butyl-[1,1'-biphenyl]-4-yl)-*N*-(4-(4,4,5,5-tetramethyl-1,3,2-dioxaborolan-2–yl)phenyl)-[1,1'–biphenyl]-4-amine (11). In a Schlenk tube compound **14** (300 mg, 0.510 mmol, 1.0 eq.), $PdCl_2(CN)_2$ (3.89 mg, 0.015 mmol, 3 mol%.), and SPhos (12.3 mg, 0.030 mmol, 6 mol%) were added then evacuated and nitrogen atmosphere established. Then dry 1,4–dioxane (2 mL) was add and stirred at r.t before adding 4,4,5,5-tetramethyl-1,3,2-dioxaborolane (0.155 mL, 137 mg, 1.07 mmol, 2.1 eq.) and Et_3N (0.220 mL,160 mg, 1.58 mmol, 4.0 eq.). The reaction mixture was stirred for overnight at 110 °C. After cooling to r.t the reaction mixture was poured into water (100 mL). The aqueous layer was extracted with DCM (3x15 mL). The combined organic extracts were dried over Na_2SO_4, filtered and concentrated under reduced pressure to give crude compound **11**, which was used without further purification.

DFT calculations

Table S1. Optimized structure and electron distribution in HOMO and LUMO levels of **D1** to **D5** dyes. Optimization of the ground–state geometry at the density functional theory (DFT) level was carried out using the B3LYP functional and the 6–31G(d,p) basis set [1-4]. Solvent effects were introduced by using the polarized continuum model[5]. A subsequent vibrational frequency calculation was performed to ensure that the structure obtained in the first step is a true minimum. The lowest 5 excited states were then computed time–dependent density functional theory (TD–DFT) using the range–separated CAM–B3LYP functional and the 6–31G(d,p) atomic basis set[6]. All calculations were carried out using the Gaussian 09 software[7].

Structure	HOMO (eV)	LUMO (eV)
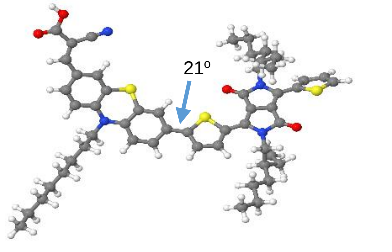 **D1**	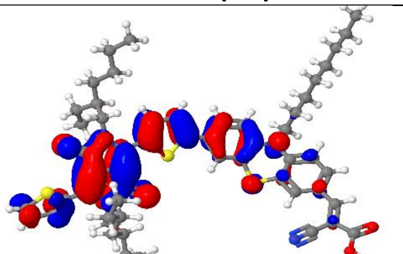 -5.05	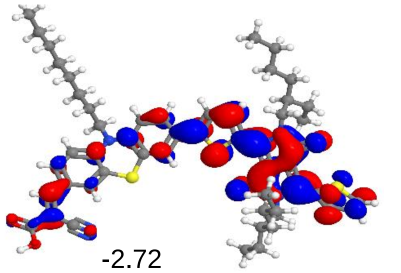 -2.72
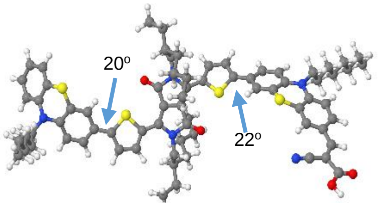 **D2**	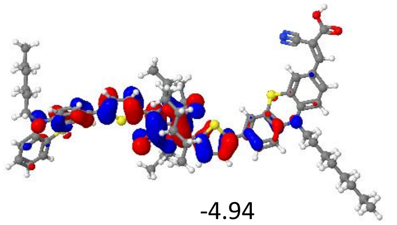 -4.94	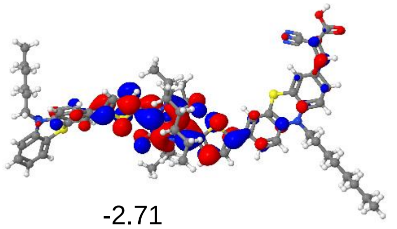 -2.71
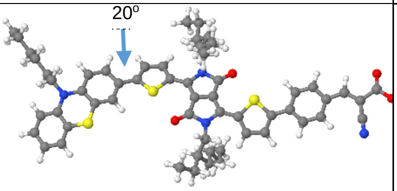 **D3**	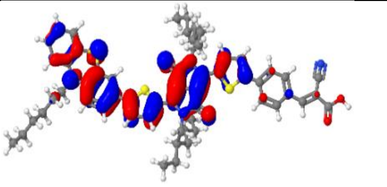 -4.91	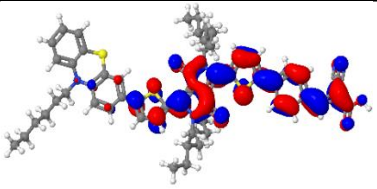 -3.01
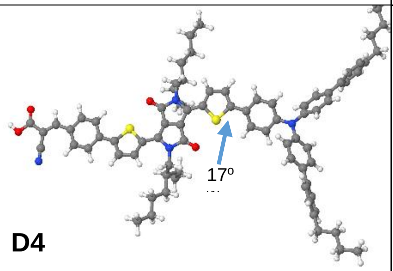 **D4**	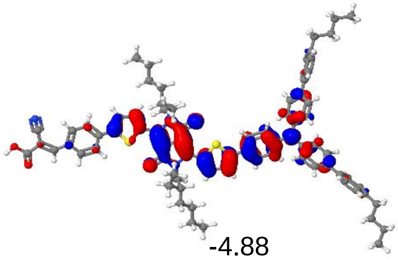 -4.88	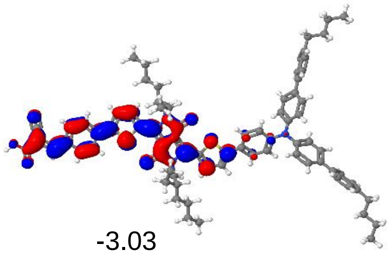 -3.03
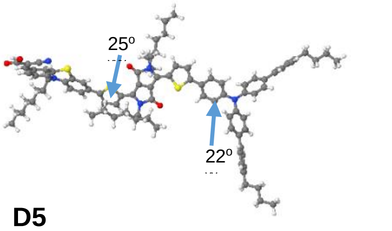 **D5**	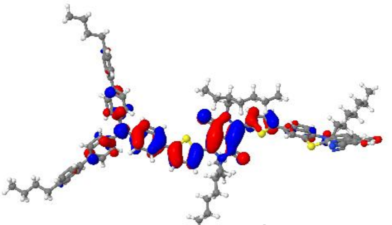 -4.85	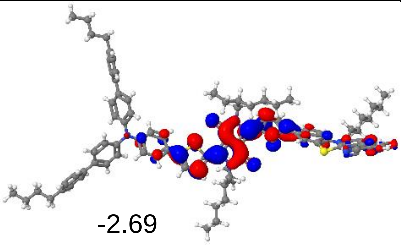 -2.69

Absorption and emission spectra of the dyes D1–D5

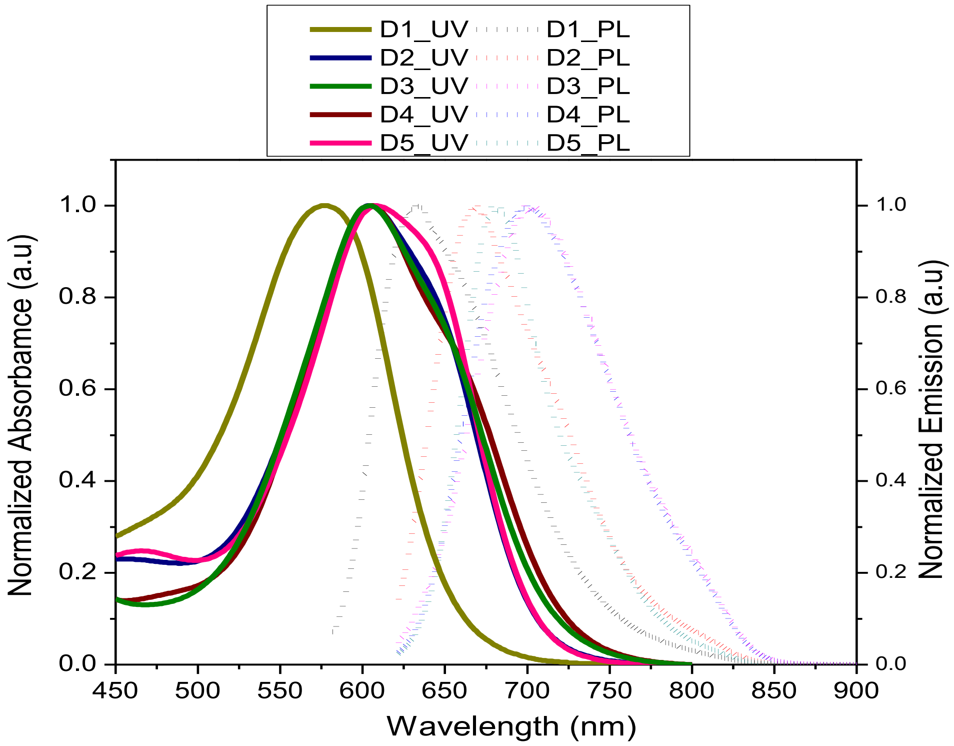

Figure S1. Normalized absorption and emission spectra of the dyes **D1**–**D5** in DCM solution.

Cyclic voltammogram of dyes D1–D5

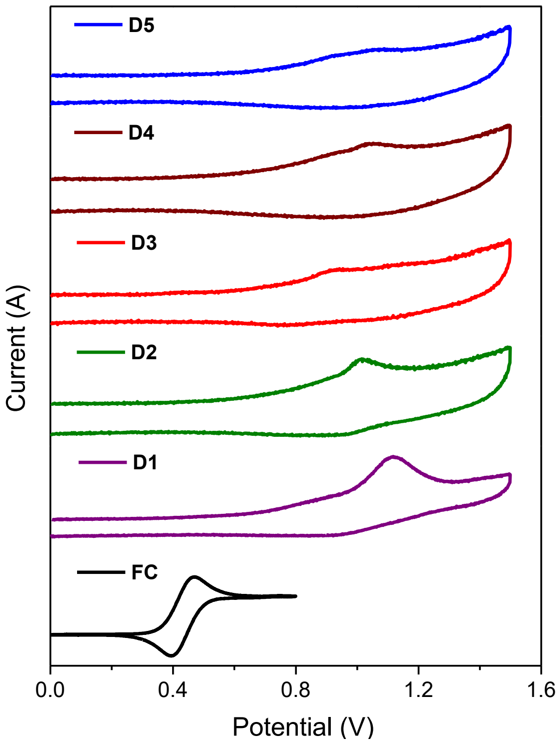

Figure S2. Cyclic voltammogram of dyes **D1–D5**. All measurements were done in solution with $TBAPF_6$ as supporting electrolyte (0.1 M in acetonitrile) at Scan rate 50 mV/s.

^{1}H and ^{13}C NMR spectra of key building blocks and dyes

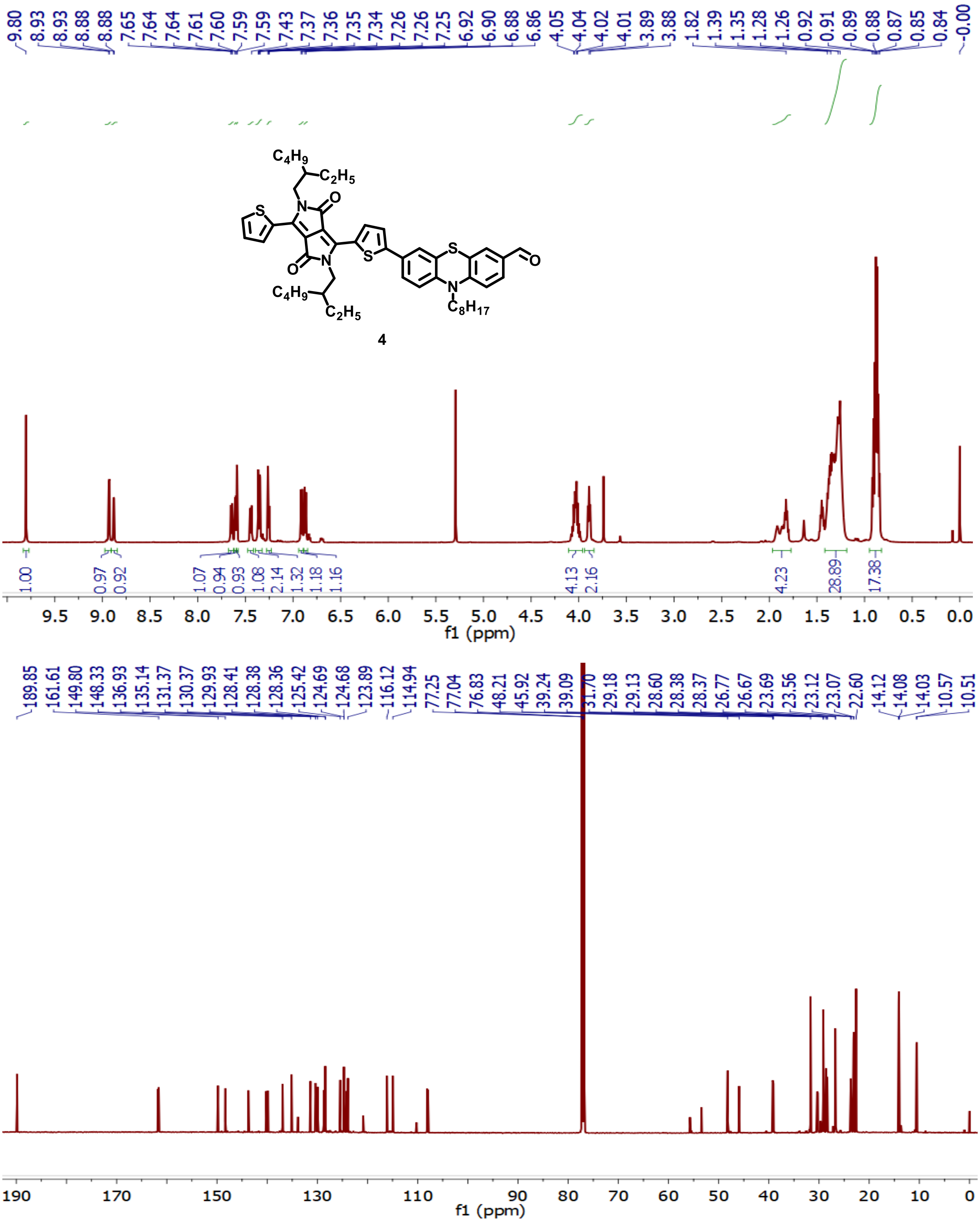

Figure S3. ^{1}H (600 MHz, $CDCl_3$) and ^{13}C NMR (150 MHz, $CDCl_3$) spectra for **4**.

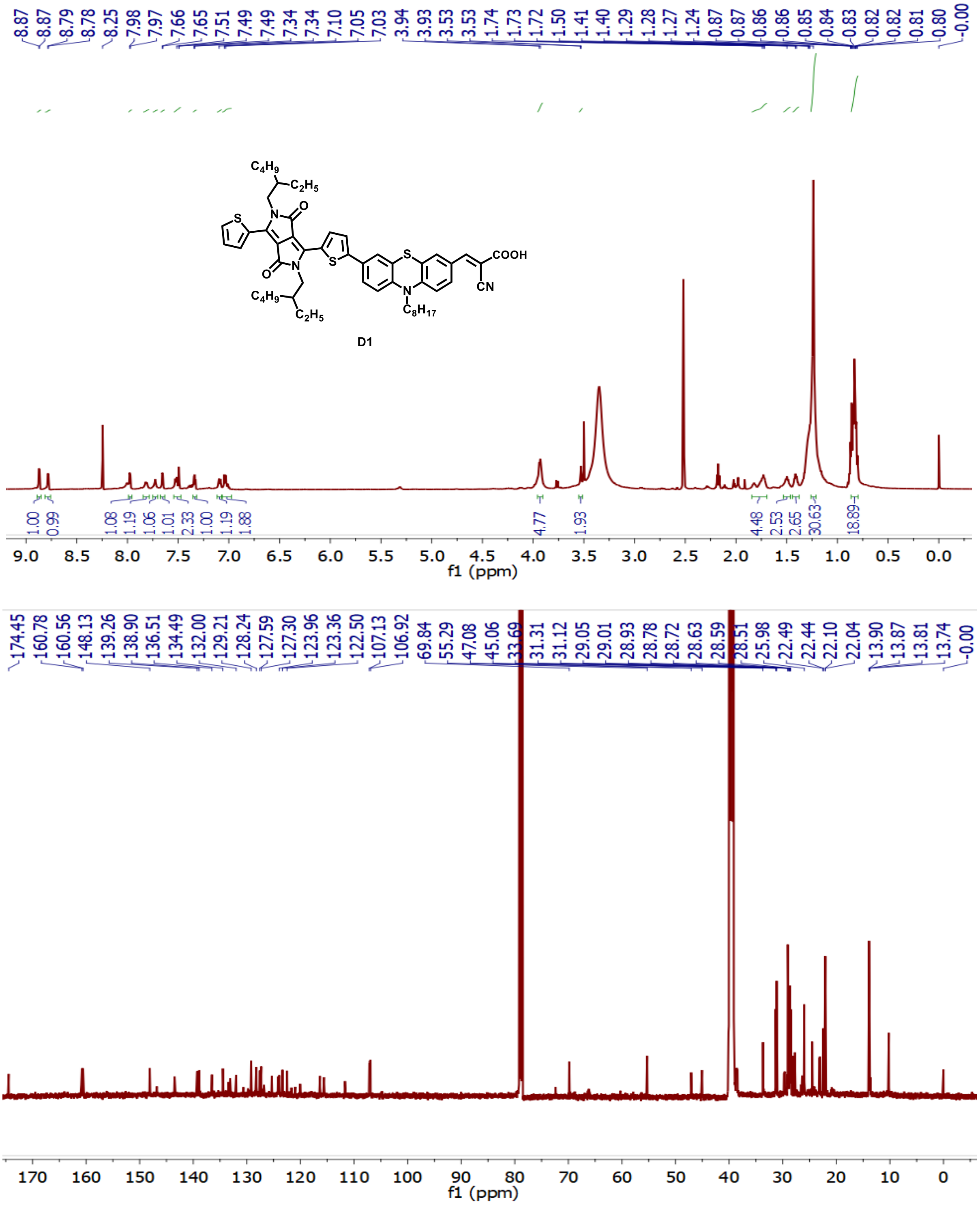

Figure S4. ^{1}H [600 MHz, DMSO-d_6 : $CDCl_3$ (4:1 v/v)] and ^{13}C NMR [150 MHz, DMSO-d_6 : $CDCl_3$ (4:1 v/v)] spectra for **D1**.

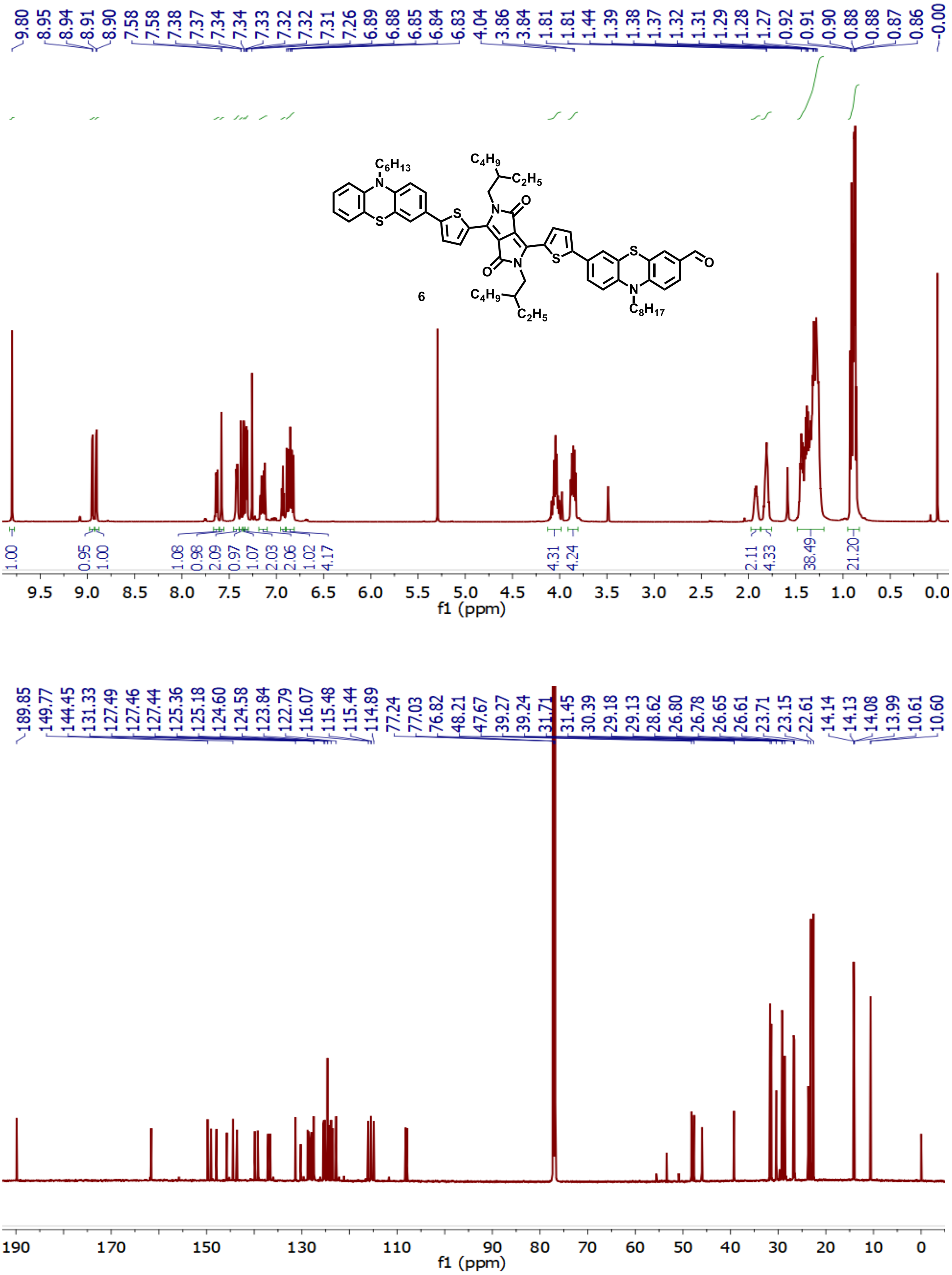

Figure S5. ^{1}H (600 MHz, $CDCl_3$) and ^{13}C NMR (150 MHz, $CDCl_3$) spectra for **6**.

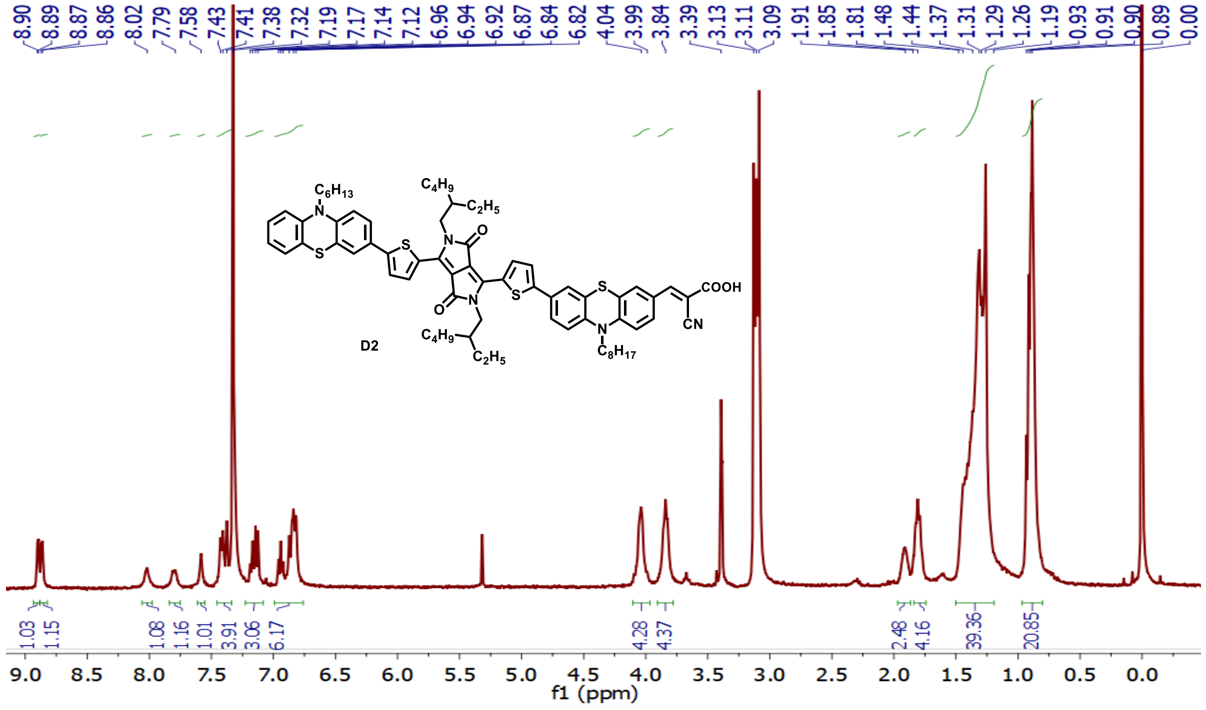

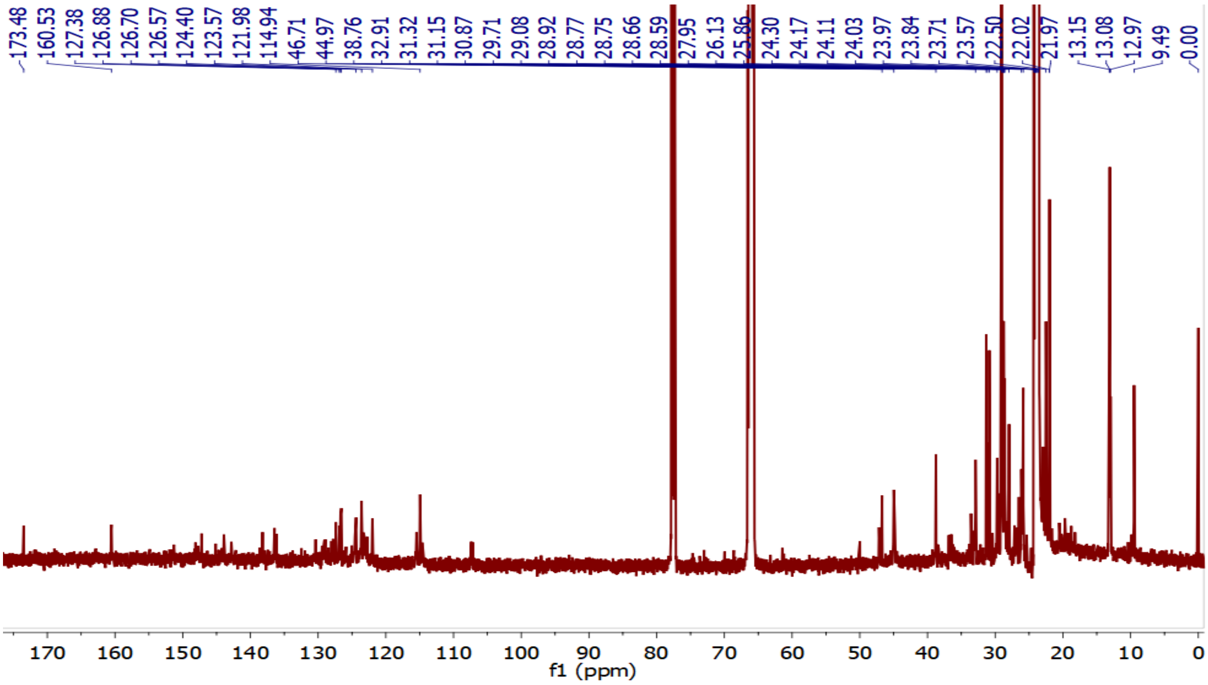

Figure S6. ^{1}H [400 MHz, $CDCl_3$: CD_3OD (4:1 v/v)] and ^{13}C NMR [150 MHz, DMSO-d_6 : $CDCl_3$ (4:1 v/v)] spectra for **D2**.

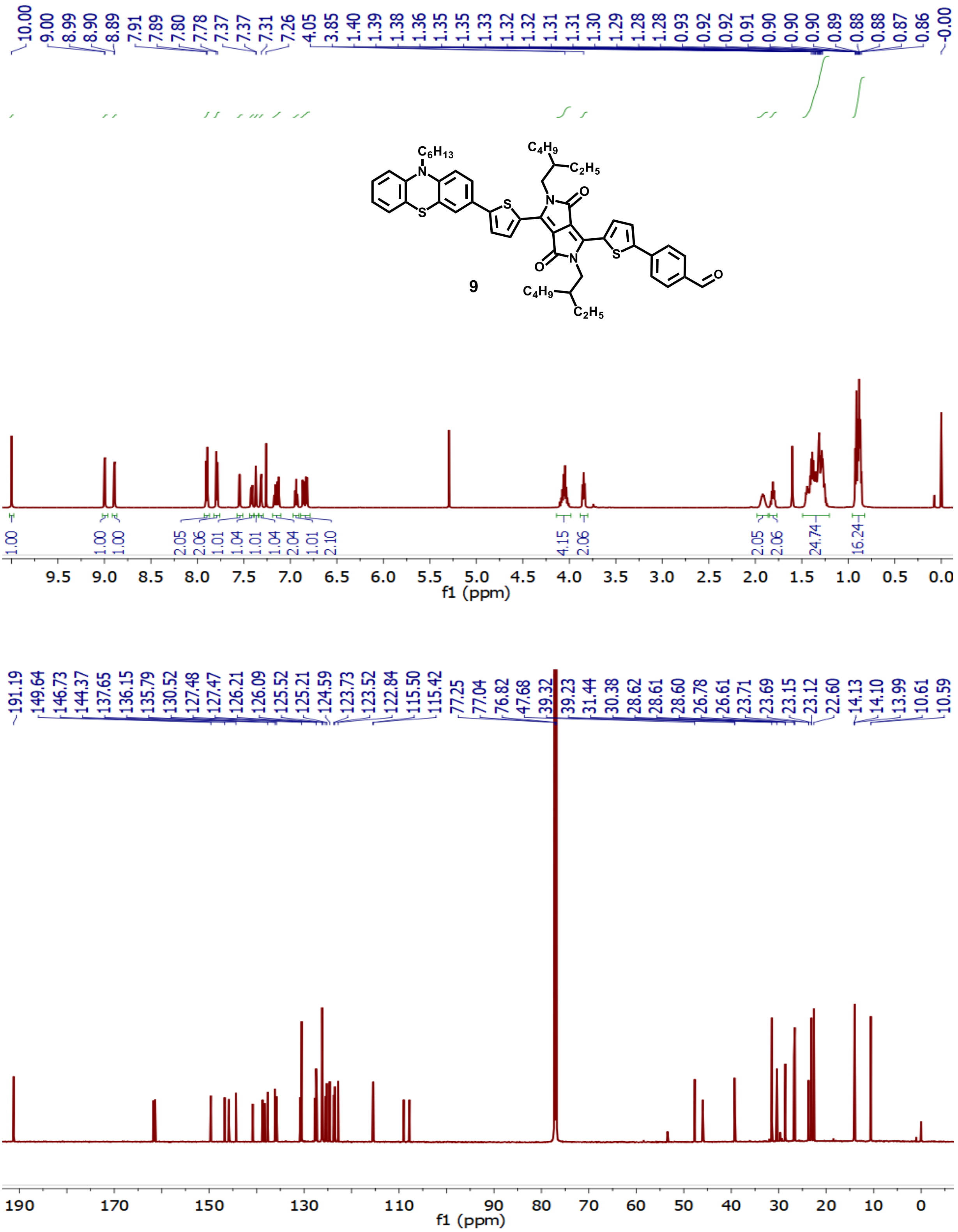

Figure S7. ^{1}H (600 MHz, $CDCl_3$) and ^{13}C NMR (150 MHz, $CDCl_3$) spectra for **9**.

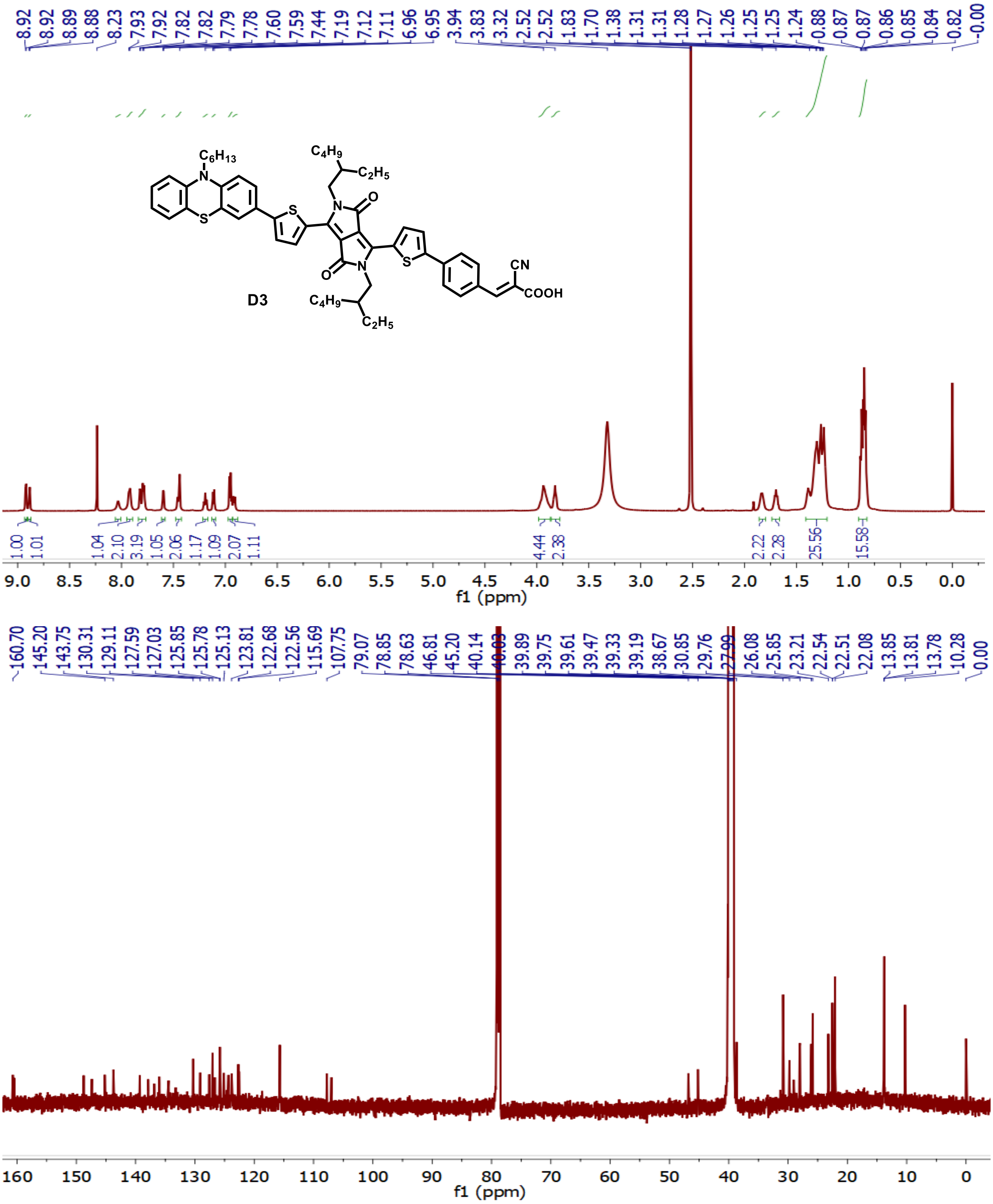

Figure S8. ^{1}H [600 MHz, DMSO-d_6 : $CDCl_3$ (4:1 v/v)] and ^{13}C NMR [150 MHz, DMSO-d_6 : $CDCl_3$ (4:1 v/v)] spectra for **D3**.

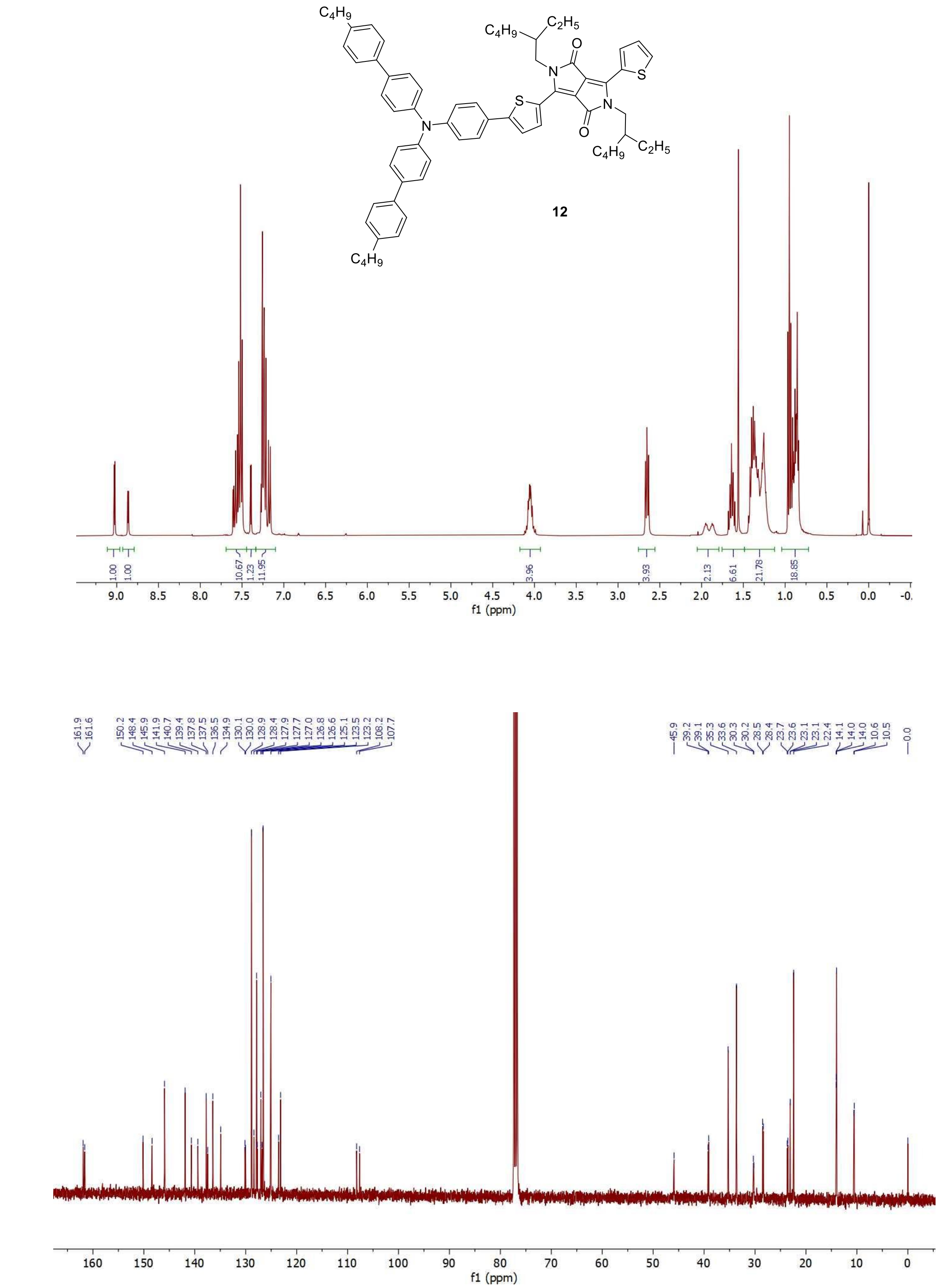

Figure S9. ^{1}H (400 MHz, $CDCl_3$) and ^{13}C NMR (100 MHz, $CDCl_3$) spectra for **12**.

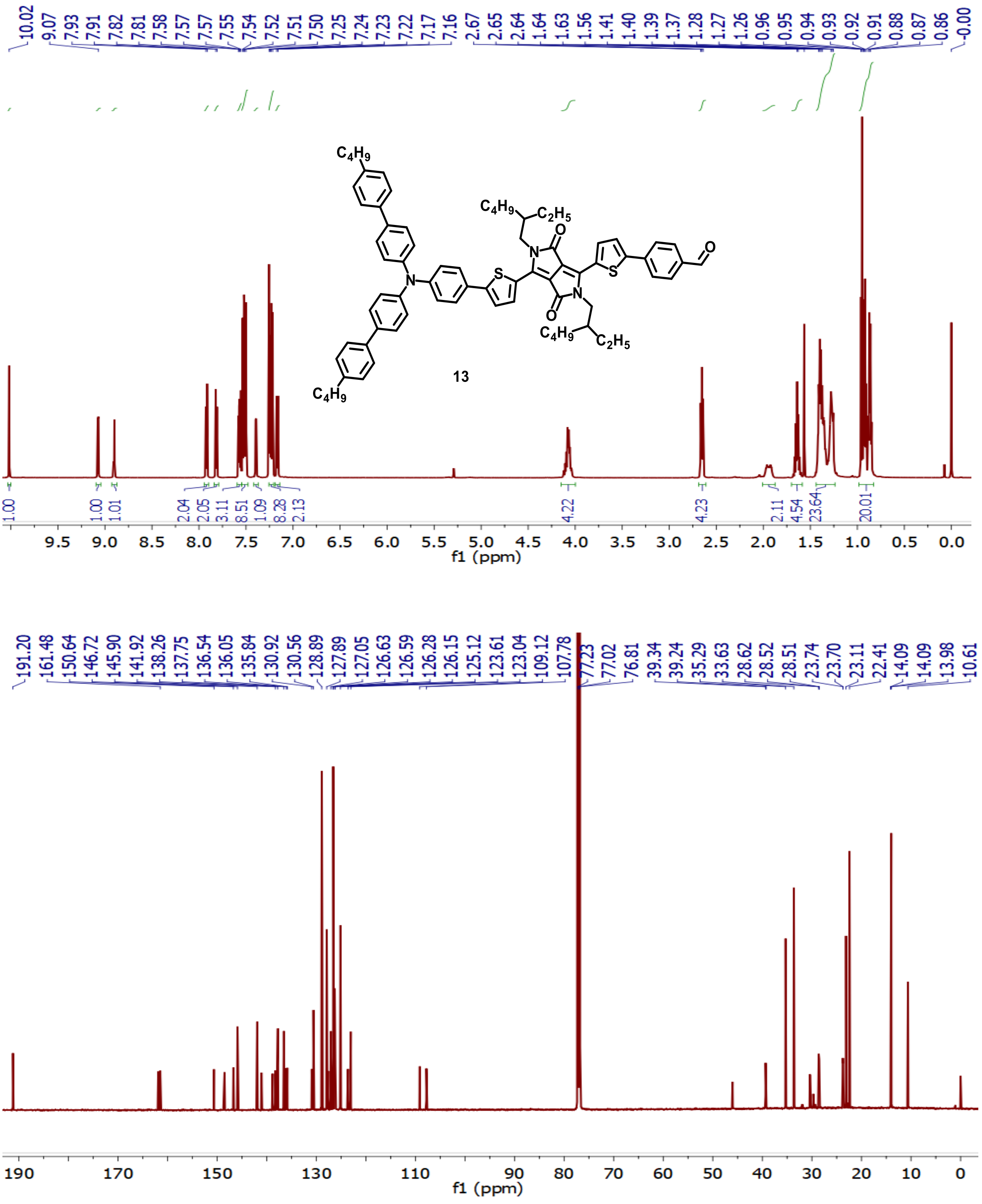

Figure S10. ^{1}H (600 MHz, $CDCl_3$) and ^{13}C NMR (150 MHz, $CDCl_3$) spectra for **13**.

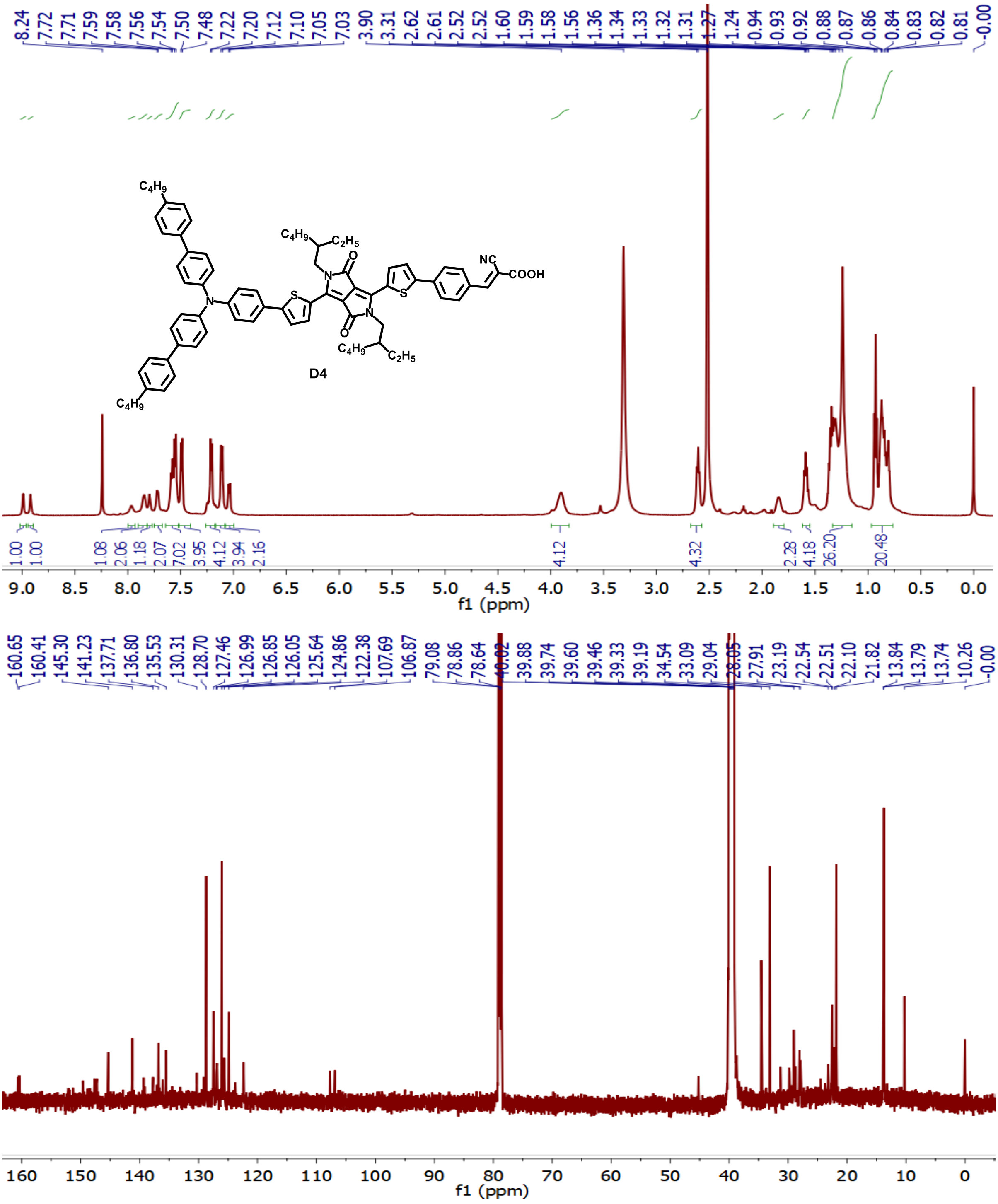

Figure S11. ^{1}H [600 MHz, DMSO-d$_6$: $CDCl_3$ (4:1 v/v) and ^{13}C NMR [150 MHz, DMSO-d$_6$: $CDCl_3$ (4:1 v/v)] spectra for **D4**.

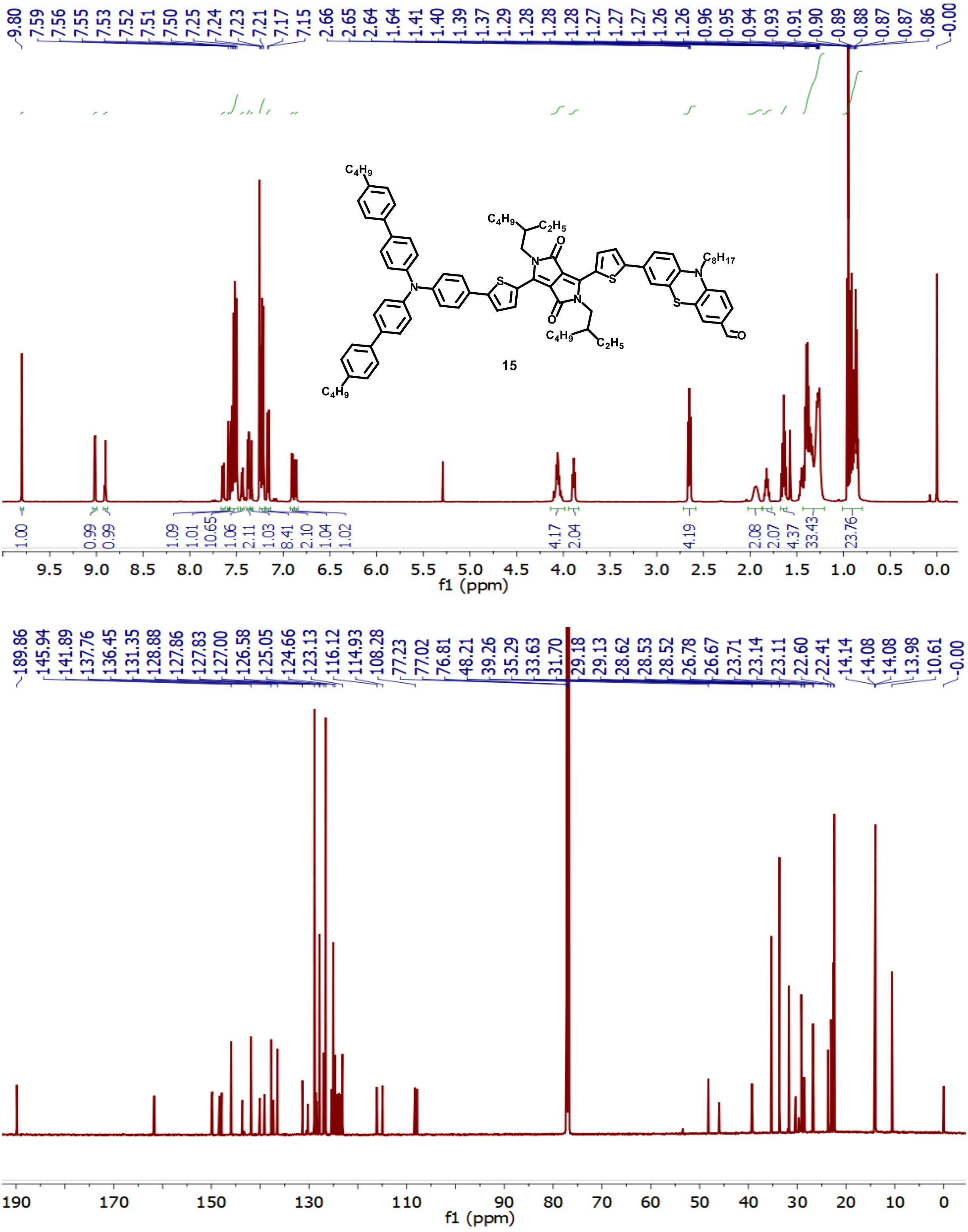

Figure S12. ^{1}H (600 MHz, $CDCl_3$) and ^{13}C NMR (150 MHz, $CDCl_3$) spectra for **15**.

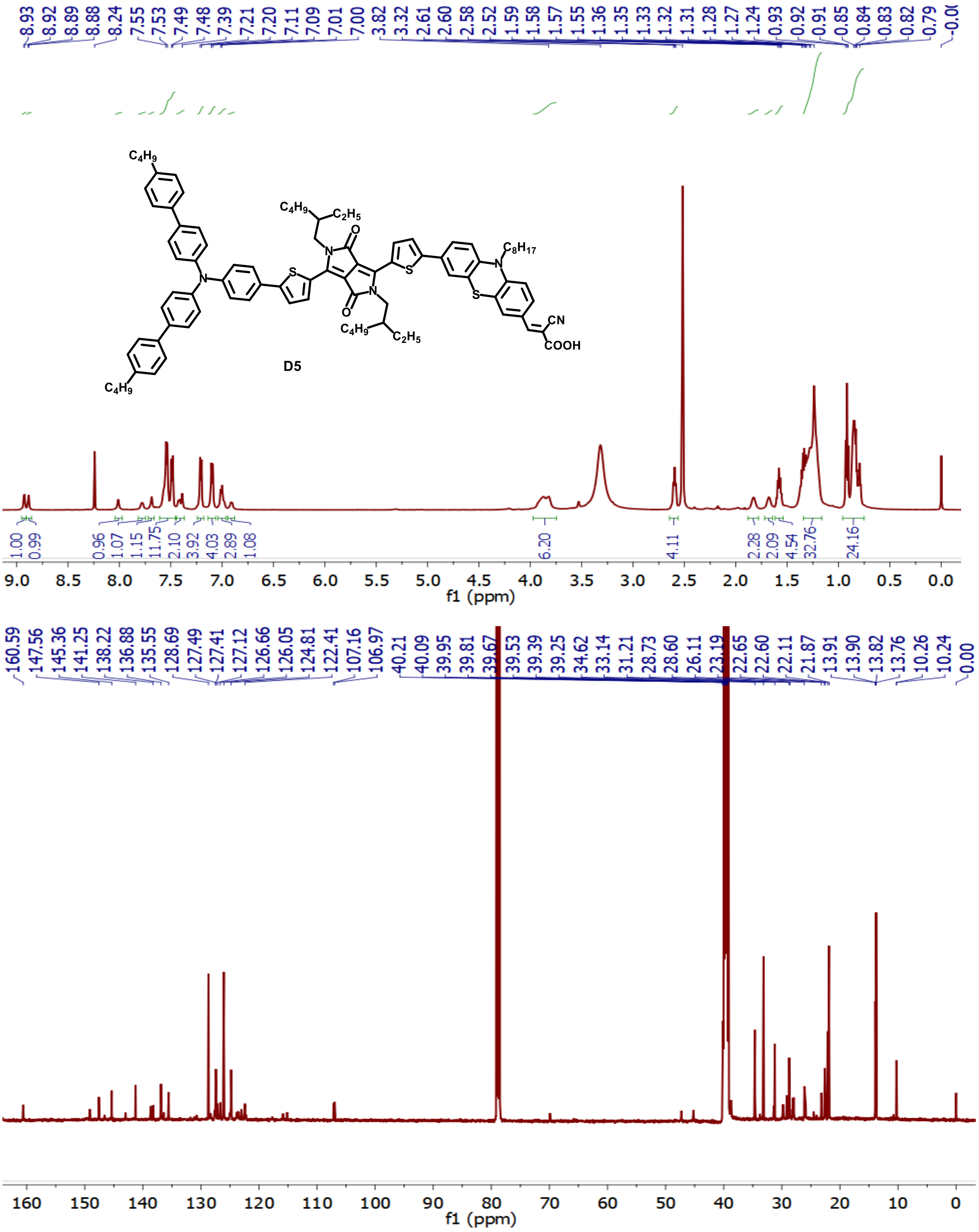

Figure S13. ^{1}H [600 MHz, DMSO-d_6: $CDCl_3$ (4:1 v/v)] and ^{13}C NMR [150 MHz, DMSO-d_6 : $CDCl_3$ (4:1 v/v)] spectra for **D5**.

References

1. Becke, A. D., Density-functional thermochemistry. III. The role of exact exchange. *J. Chem. Phys.* **1993**, 98, 5648-5652.
2. Lee, C.; Yang, W.; Parr, R. G., Development of the Colle-Salvetti correlation-energy formula into a functional of the electron density. *Phys. Rev. B: Condens. Matter* **1988**, 37, 785-789.
3. Stephens, P. J.; Devlin, F. J.; Chabalowski, C. F.; Frisch, M. J., Ab Initio calculation of vibrational absorption and circular dichroism spectra using density functional force fields. *J. Phys. Chem.* **1994**, 98, 11623-11627.
4. Vosko, S. H.; Wilk, L.; Nusair, M., Accurate spin-dependent electron liquid correlation energies for local spin density calculations: a critical analysis. *Can. J. Phys.* **1980**, 58, 1200-1211.
5. Tomasi, J.; Mennucci, B.; Cammi, R., Quantum mechanical continuum solvation models. *Chem. Rev.* **2005**, 105, 2999-3093.
6. Yanai, T.; Tew, D. P.; Handy, N. C., A new hybrid exchange-correlation functional using the Coulomb-attenuating method (CAM-B3LYP). *Chem. Phys. Lett.* **2004**, 393, 51-57.
7. Gaussian 09, R. A., M. J. Frisch, G. W. Trucks, H. B. Schlegel, G. E. Scuseria, M. A. Robb, J. R. Cheeseman, G. Scalmani, V. Barone, G. A. Petersson, H. Nakatsuji, X. Li, M. Caricato, A. Marenich, J. Bloino, B. G. Janesko, R. Gomperts, B. Mennucci, H. P. Hratchian, J. V. Ortiz, A. F. Izmaylov, J. L. Sonnenberg, D. Williams-Young, F. Ding, F. Lipparini, F. Egidi, J. Goings, B. Peng, A. Petrone, T. Henderson, D. Ranasinghe, V. G. Zakrzewski, J. Gao, N. Rega, G. Zheng, W. Liang, M. Hada, M. Ehara, K. Toyota, R. Fukuda, J. Hasegawa, M. Ishida, T. Nakajima, Y. Honda, O. Kitao, H. Nakai, T. Vreven, K. Throssell, J. A. Montgomery, Jr., J. E. Peralta, F. Ogliaro, M. Bearpark, J. J. Heyd, E. Brothers, K. N. Kudin, V. N. Staroverov, T. Keith, R. Kobayashi, J. Normand, K. Raghavachari, A. Rendell, J. C. Burant, S. S. Iyengar, J. Tomasi, M. Cossi, J. M. Millam, M. Klene, C. Adamo, R. Cammi, J. W. Ochterski, R. L. Martin, K. Morokuma, O. Farkas, J. B. Foresman, and D. J. Fox, Gaussian, Inc., Wallingford CT, 2016.
